# Supplementary material for: Memristor-based adaptive neuromorphic perception in unstructured environments
Source: Nat Commun. 2024 May 31;15:4671. doi: 10.1038/s41467-024-48908-8 (PMC11143376; doi:10.1038/s41467-024-48908-8)
Supplement: Supplementary file 1 — Supplementary Information [file 41467_2024_48908_MOESM1_ESM.docx]

# Supplementary Information

**Memristor-based adaptive neuromorphic perception in unstructured environments**

**Authors**

Shengbo Wang^†1^, Shuo Gao^†*1^, Chenyu Tang^2^, Edoardo Occhipinti^3^, Cong Li^1^, Shurui Wang^1^, Jiaqi Wang^1^, Hubin Zhao^4^, Guohua Hu^5^, Arokia Nathan^6^, Ravinder Dahiya^7^, Luigi Giuseppe Occhipinti^*2^

**Affiliations**

^1^School of Instrumentation and Optoelectronic Engineering, Beihang University, Beijing, China

^2^Department of Engineering, University of Cambridge, Cambridge, UK

^3^UKRI Centre for Doctoral Training in AI for Healthcare, Department of Computing, Imperial College London, UK

^4^HUB of Intelligent Neuro-engineering (HUBIN), CREATe, Division of Surgery and Interventional Science, UCL, HA7 4LP, Stanmore, UK

^5^Department of Electronic Engineering, The Chinese University of Hong Kong, Shatin, N. T., Hong Kong S. A. R., China

^6^Darwin College, University of Cambridge, Cambridge, UK and School of Information Science and Engineering, Shandong University, Qingdao 266237, China

^7^Bendable Electronics and Sustainable Technologies (BEST) Group, Department of Electrical and Computer Engineering, Northeastern University, Boston, MA 02115, USA

^†^These authors contributed equally to this work

^*^Correspondence to: shuo_gao@buaa.edu.cn, lgo23@cam.ac.uk

Table of Contents

[Supplementary Information 1](#_Toc166585992)

[Discussion 5](#_Toc166585993)

[1. Requirements for robotics 5](#_Toc166585994)

[2. The challenges of unstructured working environments and an overview of the current research 6](#_Toc166585995)

[**2.1 The definition of unstructured working environments** 6](#_Toc166585996)

[**2.2 Detailed challenges of grasping and autodriving** 6](#_Toc166585997)

[**2.3 Current research** 7](#_Toc166585998)

[3. Differential processing of sensory information in biology and its advantages. 9](#_Toc166585999)

[**3.1** **The structure of biological sensory systems** 9](#_Toc166586000)

[**3.2** **Differential processing of sensory information** 9](#_Toc166586001)

[**3.3** **The sensory processing model** 10](#_Toc166586002)

[**3.4** **Advantages** 10](#_Toc166586003)

[4. Mathematical proof of the advantages of differential neuromorphic computing 10](#_Toc166586004)

[5. Modulation schemes for memristors in differential neuromorphic computing 11](#_Toc166586005)

[6. Adaptation based on external sensory information 12](#_Toc166586006)

[7. Implementation of a biological tactile sensory system based on memristors 13](#_Toc166586007)

[**7.1** **Differential processing in biology** 13](#_Toc166586008)

[**7.2** **Implementation based on memristors** 14](#_Toc166586009)

[8. Biological visual sensory systems and memristor-based implementation 15](#_Toc166586010)

[**8.1** **Biological differential processing** 16](#_Toc166586011)

[**8.2** **Implementation based on memristors** 16](#_Toc166586012)

[9. The multisensory scalability of this memristor-assisted method of perception 17](#_Toc166586013)

[**9.1** **The principles of processing multisensory information** 17](#_Toc166586014)

[**9.2** **Outlook** 17](#_Toc166586015)

[10. The use of nociceptors for amplification in grasping applications 18](#_Toc166586016)

[11. Comparison of the use of memristors 18](#_Toc166586017)

[12. Comparison between our methods and PID control 19](#_Toc166586018)

[13. Explanation of the proposed method of differential processing 19](#_Toc166586019)

[14. Comparison between the memristor-assisted visual perception approach and dynamic vision system 20](#_Toc166586020)

[Supplementary Figures 22](#_Toc166586021)

[Fig S1. The structure and properties of the piezoresistive film. 22](#_Toc166586022)

[Fig S2. The switching mechanisms of the self-directed channel (SDC) memristor. 23](#_Toc166586023)

[Fig S3. The control circuit for the memristor. 24](#_Toc166586024)

[Fig S4. The main system structure. 25](#_Toc166586025)

[Fig S5. The system background noise test. 26](#_Toc166586026)

[Fig S6. The different modulation types. 27](#_Toc166586027)

[Fig S7. Tactile stimuli differential processing by biological receptors. 28](#_Toc166586028)

[Fig S8. Other differential processing functions. 29](#_Toc166586029)

[Fig S9. Processing functions in dynamic scenarios. 30](#_Toc166586030)

[Fig S10. Control logic for pain reflex and slip detection. 31](#_Toc166586031)

[Fig S11. Memristor model used in visual information processing. 32](#_Toc166586032)

[Fig S12. Noise Testing of Vision Circuits. 33](#_Toc166586033)

[Fig S13. Influence of threshold selection in visual differential processing. 34](#_Toc166586034)

[Fig S14. Impact of object distance on system detection performance. 35](#_Toc166586035)

[Fig S15. Detection of road markings. 36](#_Toc166586036)

[Fig S16. Detection of light sources. 37](#_Toc166586037)

[Fig S17. Analysis of detection performance for moving individuals. 38](#_Toc166586038)

[Fig S18. Detection for extremely hazardous scenarios. 39](#_Toc166586039)

[Fig S19. Detailed detection results in unstructured environments. 40](#_Toc166586040)

[Fig S20. Processing of temperature sensing information. 41](#_Toc166586041)

[Fig S21. Processing of humidity sensing information. 42](#_Toc166586042)

[Fig S22. The differential processing model. 43](#_Toc166586043)

[Fig S23. Comparison between other works. 44](#_Toc166586044)

[Fig S24. The switch in modulation schemes achieved by FPGA controlling. 45](#_Toc166586045)

[Fig S25. Comparison between the image captured by a standard car camera, the output from an event-based camera, and the outcomes achieved through the differential neuromorphic computing approach. 46](#_Toc166586046)

[Fig S26. Comparison between the differential neuromorphic computing and PID control. 47](#_Toc166586047)

[Supplementary Tables 48](#_Toc166586048)

[Supplementary Table 1 The modulation scheme 48](#_Toc166586049)

[Supplementary Table 2 Comparative Analysis of Visual Information Extraction Algorithm Complexities 48](#_Toc166586050)

[Supplementary Table 3 Comparative Analysis of Our Methods with Current Neuromorphic Technologies 49](#_Toc166586051)

# Discussion

1. **Requirements for robotics**

In the working process of robotics, there are three core parts: perception, processing, and execution. Among these, perception is particularly important because it provides basic raw data. However, as robotics ventures into unknown environments, building an effective perception system for these unfamiliar external environments has become an urgent problem to be solved. This is particularly important for machines designed for extreme adventures, where the accuracy of detecting external damage signals significantly impacts the quality of services rendered and their overall lifespan (*1*–*3*).

In unknown environments, robotics often interacts with the environment and objects that have unknown attributes and complex features. This requires robotics to have comprehensive perception abilities, which refers to the ability to fully understand the main information in the environment, extract the key features, and preprocess it accordingly. The method of perception should have good adaptability and generality, allowing robotics to perceive and understand information in different modalities (including vision and touch) and to switch flexibly to prelearned tasks. When the robotics lack the capacity for comprehensive perception, the key features of the object can be lost during the process of perception, resulting in poor interactions. For example, in robotic grasping, if an ~~unknown~~ object has a smooth surface and is fragile, a failure to perceive the characteristics of the smooth surface may lead to excessive force being applied during grasping, potentially damaging the object’s structure. Therefore, it is crucial for robotics to possess the capacity for comprehensive perception (*4*–*6*).

Biology possesses this comprehensive ability to perceive the external world. During the process of biological perception, various sensory receptors (such as nociceptors, fast-adapting receptors, and slow-adapting receptors) work in synergy with the neurons to extract key features from the external stimuli and carry out the corresponding preprocessing. For example, in tactile perception of information, nociceptors help organisms recognize harmful stimuli such as mechanical stress and extreme temperatures, enabling them to swiftly initiate motor reflexes. Meanwhile, receptors can adapt to milder stimuli and reduce the computational load. This method of perception is scalable, not only for tactile information but also for visual and auditory stimuli. By extracting features from the raw sensory information and differentiating them via processing through distinct neural pathways, this method allows us to adapt to the external environment in a highly generalized manner rather than being limited to specific scenes or objects.

In conclusion, this highly efficient perception mechanism serves as a valuable reference for the advancement of future robotics. We aspire to assist these robotics to meet the requirements for adaptability in comprehensive perceptual capability akin to that of living organisms, enabling technologies to better comprehend environmental information.

1. **The challenges of unstructured working environments and an overview of the current research**

Given the requisites for contemporary robots in the previous section, it is evident that these requisites primarily arise from the developmental trend of robotics. This trend extends from controlled laboratory and factory environments to include dynamic home and business environments, characterized by heightened unpredictability. These environments, often referred to as unstructured environments, present challenges to a robot's ability to perceive its surroundings. In this section, we start by exploring the definition of unstructured environments to gain a comprehensive overview of the attributes of the working environments for future robotics. Subsequently, we discuss the challenges posed by unstructured environments, focusing specifically on the tasks of grasping and autonomous driving. Furthermore, we review the current research efforts aimed at addressing the challenges associated with unstructured environments.

**2.1 The definition of unstructured working environments**

Unstructured environments refer to environments that are not prearranged or modified to facilitate a robot's execution of a task. These environments consist of various objects and interaction scenarios where the robot lacks prior knowledge of the properties of the objects and the environment. For instance, in scenarios of robotic grasping, traditional industrial robots operate in structured environments where they have prior knowledge of the objects’ properties (geometric shapes, masses, friction coefficients, etc.), whereas in unstructured environments, the contact properties of objects are completely unknown and highly variable, posing significant challenges for robots operating in these unpredictable surroundings (*7*, *8*).

**2.2 Detailed challenges of grasping and autodriving**

In this section, we discuss the impact of unstructured environments on two specific tasks: grasping and autodriving.

**2.2.1 Grasping**

In a structured environment, a robot possesses precise information about the position and shape of the object to be grasped, which can be described using a straightforward mathematical model. Consequently, the entire task of grasping benefits from well-defined modeling assumptions, which enable the robot to establish its plan of grasping with precision. Nonetheless, in unstructured environments, the variety of objects to be grasped is extensive, and their contact properties remain unknown. As a result, there is no explicit mathematical model available, rendering planning for precise grasping unfeasible in these unstructured settings. To address the uncertainties posed by unstructured environments during the process of grasping, it is crucial to enable the robot to acquire and comprehend an object’s properties. This perception needs to be robust, generalizable, and capable of sensing multiple properties of the object. Furthermore, it should assist the robot in adjusting its strategy of grasping, including the mode and the control of force, based on the learned sensory information. This adaptive approach is essential for enhancing the robot's performance in unstructured environments (*9*–*12*).

**2.2.2 Autodriving**

In structured environments, the process of driving benefits from prior knowledge, such as the expected appearance of objects and their corresponding trajectories of motion. Additionally, structured environments are more deterministic and are often characterized by explicit information about the map and the road. Conversely, there is a lack of a priori knowledge alongside high levels of uncertainty in unstructured environments, which encompass variables such as obstacles with unknown locations and the erratic movement of dynamic obstacles. The complex and uncertain nature of unstructured environments demands a strong capability for visual perception to carry out the task of autonomous driving. One crucial requirement is the swift detection and screening of dynamic obstacles and environmental information, which facilitates the system's process of decision-making, ensuring safe driving and enhancing the system’s environmental understanding (*13*–*15*).

**2.3 Current research**

When the information about the external environmental is dynamic and unstructured, perceiving and comprehensively understanding this information requires a significant amount of storage and computation. Conventional robotics operates within the von Neumann architecture of separated storage and computation. When handling numerous tasks in dynamic settings, the frequent transfer of information between the storage and computation units in von Neumann architecture leads to significant power usage and delays. These limitations become increasingly pronounced as the volume of perceived sensory data approaches human-level quantities. However, memristors, which are devices with the ability to simultaneously store and process information, are highly suitable for the real-time sensing and processing of unstructured information in unknown environments. This similarity opens up the possibility of achieving the capacity for comprehensive perception in robotics (*16*–*18*). This section reviews the work on memristor-based environmental perception in robotics.

The current methods of memristor-based tactile perception mainly focus on imitating biological receptors, particularly nociceptors (*19*–*21*). Yoon et al. implemented a biological nociceptor with a thermoelectric module, a memristor, and a resistor in series with the memristor. The thermoelectric module acted as source of the external signals, generating voltage pulses of different amplitudes depending on the external thermal stimuli (*22*). The conductance of the memristor changed with the voltage pulses, and the characteristics of the voltage observed by the resistor defined key functions of a biological nociceptor, such as ‘threshold’, ‘relaxation’, ‘no adaptation’, ‘sensitization’, and ‘cure’. Similarly, Kim et al. demonstrated a solid-state nociceptor based on a Pt/HfO_2_/TiN memristor with the functions of threshold, relaxation, allodynia, and hyperalgesia. In their design, a p-FET was considered to be analogous to the spinal cord, which amplifies the output signal of a nociceptor (*23*). However, a major limitation of these efforts is that they primarily focused on emulating individual biological receptors using a single memristor, which may result in the loss of key environmental features and limit the ability to achieve a comprehensive understanding of the environmental information.

In visual perception systems, memristors can map the information of an external image to its conductance. Similar to a tactile perception system, visual perception systems can operate by connecting light-sensitive devices to ordinary memristors. Light-sensitive devices convert optical signals to electrical signals, which can trigger the switching of memristors (*24*, *25*). However, these works primarily focused on converting visual information, and there are still gaps in how to enable robotics to comprehend the environment. In the field of neuromorphic computing (where devices are not limited to memristors), inspired by biological rod cells, Zhang et al. designed a neuromorphic system for detecting moving objects (*26*). The system operated by comparing two frames of visual information to identify disparities. However, it is not very suitable for real-world scenarios of autonomous driving. Slow background changes can introduce noise during the swift detection and screening of dynamic obstacles and environmental information. Das et al. imitated the biological lobula giant movement detector (LGMD) neuron to detect potential collisions during driving, using a blue light-emitting diode (LED) as the optical source (*27*). Through designing the stimulation program, they realized a photoreceptor that generated a similar escape response to LGMD neurons that could be used to detect collisions. However, in ~~real-world scenarios~~ driving settings, the lighting environment is intricate, and a disparity exists between the actual environment and the hypothetical environment used for detecting collisions.

~~In summary, intelligent machines still require a method that can equip them with comprehensive perception capabilities and enable them to exhibit high performance in real-world scenarios.~~

1. **Differential processing of sensory information in biology and its advantages.**

To address the current issues with memristor-based biological systems for perceiving environmental information, we first discuss the structural framework for processing unstructured environmental data in biological systems. Next, we delve into the core perceptual mechanisms involved and conduct a comparative analysis to elucidate the distinct advantages of the biological core mechanism of perception (the mechanism for differential processing of sensory information).

**3.1 The structure of biological sensory systems**

Biological sensory systems play a crucial role in gathering information from the surroundings and transmitting it to the central nervous system, enabling behavioral and physiological responses (*28*–*30*). Commonly recognized systems include touch, vision, and hearing. These sensory systems consist of sensory neurons (sensory receptors), neural pathways, and the parts of the brain involved in sensory perception. When exposed to external stimuli, the receptors simultaneously encode and transform the stimuli into action potentials, which are then understood and processed differentially by the nervous system. After this conversion, the action potentials or signals are transmitted along dedicated neural pathways to the central nervous system. These pathways are composed of chains of neurons that relay information from one neuron to another, eventually reaching the appropriate regions of the brain for further processing. For example, visual information reaches the visual cortex, while auditory information targets the auditory cortex, and so on. Subsequently, the brain interprets and integrates the sensory input, allowing us to perceive and understand our environment. The brain’s interpretation of sensory information is defined as perception.

**3.2 Differential processing of sensory information**

The differential processing of sensory information is the most important processing mechanism for the biological perception of the information of an unstructured environment (*31*–*35*). This differential processing includes two aspects, the encoding of external sensory stimuli by the receptor neurons and the processing of the encoded information by the neural pathways. Encoding mainly refers to the specificity of the receptor neurons in response to the same stimulus; for example, the nociceptive receptor only responds to specifically dangerous stimuli. This process of differential responses is essentially the extraction of features from the current stimulus. During this process, organisms perceive the multidimensional features of an external stimulus and translate these features to different responses of the sensory neurons, which are represented as neural pulses with different characteristics. ~~utilize the differentiation of sensory neural responses to encode different features and perceive the multidimensional features of the external stimulus.~~ Subsequently, these pulses are processed by different subsequent neural pathways, which typically have different structures and synaptic connection weights, representing the subsequent different processing functions (*36*–*39*).

**3.3 The sensory processing model**

From the onset of the action of an external stimulus to its encoding into neural impulses, the model can be classified into two stages. The first stage can be envisioned as a filter stage, where the sensory stimulus is filtered on the basis of the feature selectivity of a given neuron. The second stage serves as an input–output stage, transforming the filtered input of the stimulus into output as a firing rate. Different receptor neurons initially exhibit variability in their feature selection, manifested by their ability to extract various features from the same stimulus. Subsequently, they encode the information of the input features into neural impulses based on these features. The encoding process also displays variability, primarily influenced by the properties of the receptor neurons themselves. Ultimately, the neural pathways process these encoding stimuli differently.

**3.4 Advantages**

This differential processing of sensory information enables organisms to perceive multiple features of external stimuli in unstructured environments and perform different processing functions accordingly, thus allowing them to clearly understand unknown environments. For example, in tactile perception, multiple afferents with different response characteristics are utilized to actively perceive environmental features, including the shape, the material, and the roughness of the contacting objects, providing information to support our subsequent decision-making. Similarly, in visual perception, our visual afferents exhibit different frequency responses for processing visual information, aiding in the filtration of rapidly changing objects.

1. **Mathematical proof of the advantages of differential neuromorphic computing**

Let us consider a single memristor system, where the external sensory input is denoted as $s$, the memristor conductance is denoted $G$, and the system’s output is denoted $y$. The relationship between the input and output of this system can be formalized as

$$y=h(s,G)$$

where $h$ represents the mapping function from the inputs $s$ and $G$ to the output $y$. Notably, $s$, $G$, and $y$ are all time-dependent variables. Assuming that the initial state of this system at $t_{0}$ is characterized by $s_{0}$, $G_{0}$, and $y_{0}$, and given that $h$ is sufficiently smooth (i.e., it possesses all the necessary partial derivatives), the system’s changes in the output ($\Delta y$) over time ($\Delta t$) can be determined by the changes in the sensory input ($\Delta s$) and in the memristor’s conductance ($\Delta G$) through a Taylor series expansion as follows

$$\Delta y=\frac{\partial h}{\partial s}\Delta s+\frac{\partial h}{\partial G}\Delta G+\frac{1}{2!}(\frac{\partial^{2}h}{\partial s^{2}}\Delta s^{2}+2\frac{\partial^{2}h}{\partial s\partial G}\Delta s\Delta G+\frac{\partial^{2}h}{\partial G^{2}}\Delta G^{2})+\ldots$$

where $\frac{\partial h}{\partial s}$ and $\frac{\partial h}{\partial G}$ represent the first-order partial derivatives of $y$ with respect to $s$ and $G$ at $(s_{0},G_{0})$, respectively, while $\frac{\partial^{2}h}{\partial s^{2}}$, $\frac{\partial^{2}h}{\partial s\partial G}$ and $\frac{\partial^{2}h}{\partial G^{2}}$ denote the second-order partial derivatives of $y$ with respect to $s$ and $G$ at $(s_{0},G_{0})$, respectively. Subsequent terms include the higher-order partial derivatives, each multiplied by the power of the difference from the corresponding variable and divided by the factorial of that order, creating the typical form of the Taylor series. Typically, the degree of the function $h$ is usually less than or equal to 2. For instance, in neuromorphic computing, $s$ is often translated to an applied modulation voltage using a linear transformation, with the memristor’s response current as the output. Thus, the higher-order partial derivatives in Eq. 2 are negligible, and the change in the system’s output simplifies to:

$$\Delta y=\frac{\partial h}{\partial s}\Delta s+\frac{\partial h}{\partial G}\Delta G+\frac{1}{2!}(\frac{\partial^{2}h}{\partial s^{2}}\Delta s^{2}+2\frac{\partial^{2}h}{\partial s\partial G}\Delta s\Delta G+\frac{\partial^{2}h}{\partial G^{2}}\Delta G^{2})$$

In Eq. 3, the values of the partial derivatives at $(s_{0},G_{0})$ are determined and can be considered as constants. Moreover, when $\Delta s$ is fixed, the change in the system’s output change ($\Delta y$, i.e., the processing function of this neuromorphic system), hinges on $\Delta G$. Moreover, according to the memristor’s electrical characteristics, the change in the memristor’s conductance $\Delta G$ can be expressed as

$$\Delta G=Z(\int Idt)=Z(\int m(s)dt)$$

where $I$ is the current through the memristor, and $Z$ represents the relationship between the changes in the memristor’s conductance and current, with the function $m$ representing the modulation scheme applied to the memristor on the basis of the current sensory input $s$. $Z$ is usually determined according to the specific material mechanisms and can be deemed to be a fixed function. In Eq. 4, it is observed that the modulation scheme $m(.)$ greatly affects $\Delta G$, thus deciding the system’s functional performance.

In our differential neuromorphic computing approach, we exploit the adaptability of the encoding function $m$, which varies with the nature of the sensory stimuli, enabling neuromorphic systems to emulate the complex processing functions observed in biology.

1. **Modulation schemes for memristors in differential neuromorphic computing**

The differential processing of sensory information in living beings can be conceptually divided into two computational phases. The first phase involves feature extraction and encoding of the sensory information, while the second phase involves the processing of the feature information by memristors.

In the initial phase, the output signal from the sensor may be either analog or digital. For analog sensor signals, feature extraction can be executed with analog/digital circuits, including the utilization of an analog filter to isolate high-frequency information from the current sensor or using an analog-to-digital converter for quantizing sensory data and then performing feature extraction operations. It is important to note that the use of analog circuits for feature extraction ensures real-time processing but may introduce potential disturbances during the transmission of signals. In contrast, digital circuits have the opposite characteristics. Next, for the memristors to process the extracted feature information, it is imperative to encode this information into analog signals. If the feature information is already in analog form, some preprocessing steps (such as amplification or limiting) can be performed to prepare the encoded signal for processing by the memristors in the second stage. However, if the feature information is originally in digital format, it is essential to convert it into analog signals before subjecting it to the processing steps outlined earlier.

The second stage involves processing the feature information using memristors. This stage resembles the achievement of computational functions through the memristor’s intrinsic ability to alter its resistance in response to the electrical signals generated by the features. The distinctiveness of this processing function becomes more pronounced when the nature of the stimulus varies according to the how the features are encoded. For instance, in the context of processing tactile information, stimuli associated with dangerous features are encoded as positive voltage pulses, whereas those related to mild features are encoded as negative voltage pulses. In the case of stimuli corresponding to dangerous features, the memristor's conductance increases, whereas it decreases for stimuli associated with mild features. Furthermore, given the memristor's role as a synaptic weight in transmitting sensory information, it possesses the capacity to amplify dangerous stimuli and adjust to mild ones. This capacity enables the differential processing of information.

1. **Adaptation based on external sensory information**

The synapse-like characteristics of memristors offer intriguing possibilities for adaptation based on external sensory information. In this perceptual approach, we can encode environmental feature information as voltage stimuli, leading to changes in the memristor’s resistance. Each specific state of the memristor represents the acquisition of distinctive feature information. In our demonstration of perceiving tactile information, the memristor’s resistance adjusts according to the real-time attributes of the current external tactile stimulus. The memristor's state information at each moment reflects the cumulative effect of the history of the stimulus. For instance, a low value of resistance in the memristor indicates that the robot has encountered potentially dangerous stimuli for an extended period, serving as a basis for the robot to trigger a pain reflex. This capacity for continuous adaptation enhances the robot's autonomy, particularly in unfamiliar environments (*10*).

Similarly, in the realm of processing visual information, high-frequency visual data are encoded as positive voltage pulse stimuli, with the pulses’ amplitude escalating in proportion to the degree of variation. Consequently, memristors with lower resistance values exhibit heightened sensitivity to high-frequency changes in the intensity of light within a given region. This heightened responsiveness is of paramount significance for real-time decision-making, particularly in autonomous driving scenarios. In conjunction with the extraction of information on the environmental features, memristors can proficiently acquire a multifaceted understanding of the surroundings, making them exceptionally well suited for robotics functioning in unstructured environments.

1. **Implementation of a biological tactile sensory system based on memristors**

Within living organisms, multiple receptors play a pivotal role in detecting external tactile stimuli, resulting in the perception of intricate and nuanced tactile information. In this section, we explain how organisms process and acquire different types of tactile information and demonstrate how our methodology can be applied to achieve biomimetic perception.

**7.1 Differential processing in biology**

The differential processing of tactile stimuli in organisms primarily involves two crucial functions: the amplification of dangerous stimuli and adaptation to mild stimuli. The former is achieved through the engagement of nociceptors and their corresponding neural pathways, whereas the latter is orchestrated by fast-adapting and slow-adapting receptors, along with their respective neural pathways.

Nociceptors are the receptors responsible for sensing potentially dangerous sensory stimuli in living organisms (*40*–*43*). Through the subsequent neural pathways, they determine the crucial functions, including ‘threshold’, ‘no adaptation’, ‘sensitization’ and ‘relaxation’. The ‘threshold’ refers to the condition in which the nociceptor responds only when the external tactile stimuli reach a certain threshold, which typically has dangerous characteristics. ‘No adaptation’ means that the nociceptor consistently increases the intensity of its response to strong stimuli as it senses and assimilates the information on danger in the environment. ‘Sensitization’ means that after perceiving certain dangerous stimuli, the nociceptor enters a sensitized state, further intensifying its response to dangerous stimuli. ‘Relaxation’ indicates that when the dangerous external stimuli are removed, the nociceptor returns to its normal state, reducing the level of sensitivity to external stimuli to the initial value. Based on these functions of nociceptors, organisms can swiftly detect dangerous stimuli, ensuring their safety. These functions are necessary for robotics operating in unknown environments.

In contrast, rapidly adapting receptors and slowly adapting receptors, along with their subsequent neural pathways, adapt to external stimuli by gradually reducing their levels of response (*31*, *44*, *45*). These receptors are the key components in achieving sensory adaptation, a function which primarily refers to adaptation to mild external stimuli and which involves detecting environmental information related to mild stimuli and continuously reducing the intensity of their response to such stimuli. Rapidly adapting receptors adapt to stimuli swiftly, whereas slowly adapting receptors do so at a more gradual pace. This perceptual mechanism aids in lightening the burden of processing on the central nervous system and enables the prompt detection of changes in the stimuli. For instance, when the intensity of a mild stimulus undergoes a sudden alteration, it becomes more noticeable due to prior adaptive processing. Therefore, sensory adaptation is also significantly relevant in scenarios such as the task of stable grasping in robotics.

**7.2 Implementation based on memristors**

The perception of external tactile information utilizes the current magnitude of pressure as the input stimulus, considering the memristor to be a synapse and where $M(.)$ defines the correlation between the memristor’s state and the features use to determine the appropriate modulation schemes. The function $V(.)$ for modulation of the memristor acts as a dynamic, multi-branch function based on the current sensory features $f(t)$ and the memristor’s resistance $M(x)$. This modulation is selected by the FPGA digital component. The tactile response strength is determined by multiplying the input stimulus by the current relative conductance of the memristor. The implementation of nociception and sensory adaptation is as described below.

In processing hazardous stimuli, we first evaluate whether the magnitude of the current pressure matches the criteria for dangerous features. Only when the current pressure surpasses a predefined threshold does the encoding unit generate positive voltage pulses, increasing the memristor’s conductance. This corresponds to the ‘threshold’ function of biological nociceptors, as demonstrated in the following formula

$v(t)=v_{noc}$ *for* $r(t)\subset r_{d}$, $t_{sta}<t<t_{end}$

$v(t)=0$ *otherwise*

where $v(t)$ represents the voltage stimulus applied to the memristor. If the pressure of the stimulus shows the characteristics of a hazard, $r_{d}$, which is a voltage pulse with an amplitude of $v_{noc}$and a duration from $t_{sta}$ to $t_{end}$, is generated. For continuous hazardous stimuli, the memristor’s conductance gradually increases under sustained positive voltage stimuli, thus not adapting to dangerous stimuli. The process can be represented as follows:

$$\frac{dx}{dt}=f(x,v(t))<0$$

where $x$ represents the state of the memristor. Under voltage pulses, the increase in the conductance of the memristor realizes processing of the stimulus as ‘no adaptation’. Furthermore, to realize the ‘sensitization’ function, we can draw inspiration from biology, where information from different time scales is considered simultaneously during processing. When the stimulus matches the features of hazards and the memristor’s conductance has already exceeded a certain threshold, it can be inferred that the robot has received information on a hazardous stimulus for a certain duration. At this point, we increase the amplitude of the positive voltage stimuli to further amplify the response to hazardous stimuli. When the external stimuli are removed or no longer meet the hazardous criteria, the encoding unit re-encodes the information. Upon removal of the stimuli, the encoding unit generates recovery pulses, restoring the memristor to its initial resistance, achieving the ‘recovery’ function.

The processing of mild stimuli follows a similar procedure to that of hazardous stimuli. First, we extract and analyze the characteristics of the external stimuli. When a tactile stimulus meets the criteria of being mild (the magnitude of the force falls within a certain range), the encoding unit generates negative voltage pulses, reducing the memristor's conductance. When a mild stimulus remains stable within a certain range, the memristor’s conductance decreases continuously, achieving adaptation to mild stimuli. This differential processing method allows us to adjust the speed of adaptation by modifying the features of the encoding pulses on the basis of the mildness, thereby emulating both the rapidly adapting and slowly adapting receptors. When the force is removed or no longer meets the criteria for a mild stimulus, the encoding unit re-encodes the information. Upon removal of the stimulus, the memristor is reset to its initial resistance.

This differential processing method shows potential for multifunctional processing. In addition to the aforementioned biomimetic functions, we also implemented the perception of normal functionality, which maintains the memristor’s resistance at a stable level (i.e., it neither amplifies nor adapts to external stimuli). It is evident that this method holds great potential for processing unstructured tactile information. By further designing the logic of feature extraction and the corresponding pulse codes, robots can acquire the capacity for multidimensional tactile sensing and improve their understanding of environmental information.

1. **Biological visual sensory systems and memristor-based implementation**

A biological visual system consists of sensory organs (eyes) and parts of the central nervous system (sensory cells, optic nerves, optic tracts, and the visual cortex). Through their coordinated efforts, living organisms are able to detect and interpret visual information. In this section, we provide a detailed description of how biology processes visual information, as well as its implementation in memristors.

**8.1 Biological differential processing**

In the process of human perception of visual information, incoming light enters the eye and passes through a compound lens composed of the cornea and a crystalline lens before being projected onto the retina. In the retina, two types of photoreceptor cells are involved in vision: rod cells and cone cells (*46*–*48*). These two classes of cells further transmit information to bipolar cells and ganglion cells, ultimately relaying it to the visual cortex, where visual perception occurs. Rod cells and cone cells differ in how they process light information. They differ in their sensitivity to light, with rod cells being highly sensitive to light and being able to function in dim lighting conditions, where cone cells are less light-sensitive and work in bright conditions. Additionally, the time course of a response to light is different between rods and cones: rods exhibit a sustained response to a flash of light, whereas cones show a brief response. This difference in the response time affects the temporal resolution of rods and cones: cones are more effective than rods at detecting light flickering at a high frequency and are better suited for perceiving quickly moving objects. This differential processing of information on the frequency of light is crucial for detecting rapid changes in the intensity of light and distinguishing them from slower changes. In autonomous driving scenarios, the differential processing of information on light frequency is also crucial for making timely driving decisions, leading the driver to respond appropriately.

**8.2 Implementation based on memristors**

In the proposed method, we initially use analog filters to extract the changes in the intensity of light and generate corresponding coding stimuli based on whether the frequency is high or low. Information on high-frequency changes in the intensity of light is crucial for real-time decision-making, whereas low-frequency information typically corresponds to slowly moving or stationary objects. To differentiate the frequency features of visual information, we can encode high-frequency information as positive voltage pulses and low-frequency information as negative pulses, causing corresponding changes in the memristor’s resistance. The stimuli encoded on the basis of their frequency can be represented by the following formulae

$$v(t)=m\times f_{light}+b>0$$

$$f_{light}\subset f_{high}$$

$$v(t)=n\times f_{light}+c<0$$

$$f_{light}\subset f_{low}$$

where $f_{light}$ is the frequency information of the intensity of the light; $f_{high}$ and $f_{low}$ represent high-frequency and low-frequency changes, respectively; and the remaining parameters are constant coefficients. When the memristor exhibits a low-resistance state, it has effectively perceived high-frequency stimuli in the external environment. Consequently, executing emergency obstacle avoidance maneuvers in such circumstances is a reasonable course of action. Conversely, when the memristor is in a high-resistance state, it suggests that the changes in the intensity of light within that area have been gradual or slow.

1. **The multisensory scalability of this memristor-assisted method of perception**

By mimicking the intrinsic nature of low-level human perception mechanisms in electronic neural circuits based on memristors, the proposed method has the potential to process multimodal sensory information. Here, we discuss the principles of this method of processing multisensory information and its potential applications.

**9.1 The principles of processing multisensory information**

Expanding the method of perception from a single sensory stimulus to multisensory information involves adjustment of the front-end sensors to accommodate different types of physical stimuli. After obtaining the raw perceptual information through sensors, feature extraction and differential processing can still be applied to this sensory information, regardless of the original nature of the physical stimuli. This scalability of perception is rooted in the fact that differential processing of sensory information is a fundamental sensory mechanism in biology, existing across multiple senses and capable of handling stimuli with varying properties and quantities. In our proposed method, when the nature of external stimuli changes and the corresponding sensors are replaced, some modules can be fine-tuned (e.g., through adjusting the amplitude of the voltage pulse generated by the encoding module) but the overall perceptual structure remains unchanged.

**9.2 Outlook**

Through leveraging the scalability of our proposed memristor-assisted method of perception, robotics can achieve comprehensive perception across diverse sensory inputs. When combined with various sensors, these robotics can better comprehend environmental information through senses including touch, vision, and hearing, among others. Such capabilities for perception can empower robotics to excel in unknown environments by understanding the features of various objects in the environment, addressing critical tasks such as path planning, object recognition, and grasping. Moreover, through the power of differential neuromorphic computing, robotics can acquire human-like sensory abilities, allowing them to interact more naturally with humans. This would include the ability to perceive and convey information through speech, gestures, facial expressions, and sound, resulting in a better understanding of and responsiveness to human needs. Furthermore, as robotics continue to evolve, they may exhibit creative capabilities. Art and creativity frequently hinge on the ability to perceive the world and grasp its intricacies. When robots have the capacity for comprehensive perception across multiple senses, including visual, auditory, tactile, olfactory, and gustatory information, they will gain access to a wealth of input data, which become instrumental in observing and understanding the surrounding environment, as well as human behaviors and emotions. As a result, robots would have access to a wider range of materials and background information to fuel their artistic and creative endeavors. Finally, robotics holds the potential to surpass human capabilities. Combined with the Turing machine, robotics not only possess efficient and sophisticated sensory capabilities akin to humans but can also quantitatively process sensory information to achieve virtually any computational functions, thereby surpassing the limitations of human ability.

**10. The use of nociceptors for amplification in grasping applications**

For humans, nociceptors play a vital role in interacting with the environments by protecting us against dangerous stimuli, suggesting their utility in robotics for increasing safety (*49*–*51*). Specifically for grasping tasks, nociceptors facilitate the safe establishment of contact through amplifying dangerous stimuli. For example, Ravinder et al. developed a neuromorphic e-skin to help robots interact with environments, with a novel design emulating nociceptors to achieve a pain reflex (*11*). Mathews et al. demonstrated a neuromorphic nociceptor based on a self-healing material that allowed a sensorized robotic arm to enhance the response to noxious stimuli and trigger a motor response immediately to avoid potential physical damage (*52*). To enhance the safety of robotic grasping in unstructured environments, we present the use of memristor-based nociceptors for detecting and amplifying noxious stimuli.

It is worth noting that nociceptor-based amplification is a consequence of the model’s design rather than a necessity arising from the use of memristors. The synapse-like characteristics of memristors bear a foundational resemblance to biological receptors, allowing for diverse implementations of memristor-based receptors, such as slow-adapting receptors, fast-adapting receptors, etc.; nociceptors are merely one kind.

**11. Comparison of the use of memristors**

Previous methodologies, such as the memristor-based nociceptors depicted in Figure S23a,d, were designed exclusively to process dangerous stimuli. This kind of design restricts the capability of the memristor to process stimuli of another nature (e.g., mild ones). Figure S23h also shows that the response of the output frequency shifts only when the input voltage surpasses a certain threshold. However, given the high similarity between memristors and biological synapses, a memristor has the ability to replicate the diverse synaptic plasticity, thus effectively emulating the intrinsic characteristics of different receptors and sensory neurons. Hence, we leveraged this capability in our work by using a memristor as a synapse in dynamic computing. As shown in Figure 1d, the scheme of modulation was selected, enabling the memristor to process and adapt to varying external sensory stimuli.

**12. Comparison between our methods and PID control**

As illustrated in Figure R26, although the differential neuromorphic computing method and PID control share the fundamental principle of smartly adjusting the outputs in response to feedback, they diverge significantly in the process of data manipulation. Specifically, PID systems focus on automatically applying an accurate responsive correction $v_{2}(t)$ to a control function on the basis of the error value $e(t)$, and the relationship between $v_{2}(t)$ and $e(t)$ can be expressed as:

$$v_{2}(t)=K_{p}e(t)+K_{i}\int_{0}^{t} e(\tau)d\tau+K_{d}\frac{de(t)}{dt}$$

where $K_{p}$, $K_{i}$, and $K_{d}$ are all non-negative and denote the coefficients for the proportional, integral, and derivative terms, respectively. In contrast, our method focuses on automatically selecting an appropriate scheme of modulation $v_{1}(t)$ allowing the memristors to process sensory stimuli, and this process can be expressed as

$$v_{1}(t)=V(f(t),M(x(t-1)))$$

where $f(t)$ represent the features extracted from the sensory input, $x(t-1)$ is the memristor’s state after the previous modulation, $M$ is the eigenvalue used for updating the memristor’s weight and is associated with its state, and $V$ is the function used for selecting the scheme. Note that, in our system, $M(x(t-1))$ represents the memristor’s resistance after the previous modulation. Thus a comparison of the two equations shows that the method presented here leverages the nonlinear characteristics of the memristor and a dynamic selection scheme for the manipulation of more complex data than the linear coefficient-based error correction used in PID. Additionally, the intrinsic memory function of memristors in our system enables real-time adaptation to changing environments. This represents a significant advantage compared with the static configuration of the parameters in PID systems.

**13. Explanation of the proposed method of differential processing**

The proposed method of differential processing in our work is a method of computation involving multi-branch functions that emulate biological sensory processing. Specifically, in our method, the memristor is automatically modulated into various states to handle different kinds of pressure or visual information. This methodology effectively utilizes the nonlinear state-space of the memristor to achieve neuromorphic-style adaptation to external sensory stimuli, as shown in Figure 1d. This stands in contrast to the state-of-the-art (SOTA) neuromorphic sensors, where ‘differential processing’ refers to detection of the changes in the sensory stimuli, as shown in Figure S25, which is a sub-function of our proposed method.

If we take the event-based sensor as an example, the concept of differential processing refers to the detection of changes in the intensity of light rather than the static intensity of light across the image. By comparison, first, our method can process both static and dynamic signals. For instance, in tasks involving the task of grasping an object, static sensory signals indicating harm are used to modulate the memristor into a nociceptor state, demonstrating the versatility of our approach. Second, when dealing with dynamic signals, we consider both the immediate changes in the sensory information and the historical data stored by the memristor, which eventually results in the abstraction of the moving object and an indication its direction of movement through the use of afterimages. The differences are further highlighted by the comparative images in Figure S25.

**14. Comparison between the memristor-assisted visual perception approach and dynamic vision system**

Artificial visual systems are designed to have a wide dynamic range, high temporal resolution, and efficient scene understanding ability. Visual sensors, as the initial sensory part, play a crucial role in these systems(*53*–*55*). Conventional frame-based cameras, like digital cameras, have the capability to capture visual information about target objects faithfully but generate lots of redundant data(*54*). This redundancy necessitates local preprocessing to make visual data more efficient, a requirement that aligns well with the capabilities of neuromorphic devices(*56*–*58*).

In our memristor-assisted visual system, the memristor array processes the light intensity changes directly at the detection point. This local processing highlights crucial information for decision-making and preserves afterimages that maintain historical visual context, filtering the background beside the moving object, making the visual data more efficient. This capability holds the potential to help with decision-making in real-world scenarios involving rapid movements. Furthermore, the system’s reaction can be customized by adjusting the memristive modulation rules according to specific sensory input characteristics, meeting the requirements for different application scenarios. For instance, tuning the amplitude of negative pulses related to low-frequency information in the demonstrated visual systems can change the duration for which afterimages are maintained, providing flexibility in response according to the scenario’s demands. Afterimages with longer duration are advantageous for tracking and predicting a single moving object, particularly when it is possibly obstructed by an obstacle. Conversely, when tracking and predicting multiple moving objects, longer afterimage duration could negatively impact accuracy due to overlapping, making shorter afterimage duration necessary.

Compared with dynamic vision cameras(*59*–*61*), our memristor-assisted approach faces the inherent limitations of frame-based cameras, including reduced performance under low-light conditions and a narrower dynamic range. In contrast, dynamic vision cameras offer advantages in capturing changes in the scene with higher temporal resolution, broader dynamic range, and lower power requirements. However, dynamic vision cameras typically lack the ability to directly generate afterimages that contain crucial temporal information. To obtain this kind of temporal information, dynamic vision cameras require additional computational resources and storage capacity.

# Supplementary Figures

## **Fig S1. The structure and properties of the piezoresistive film.**


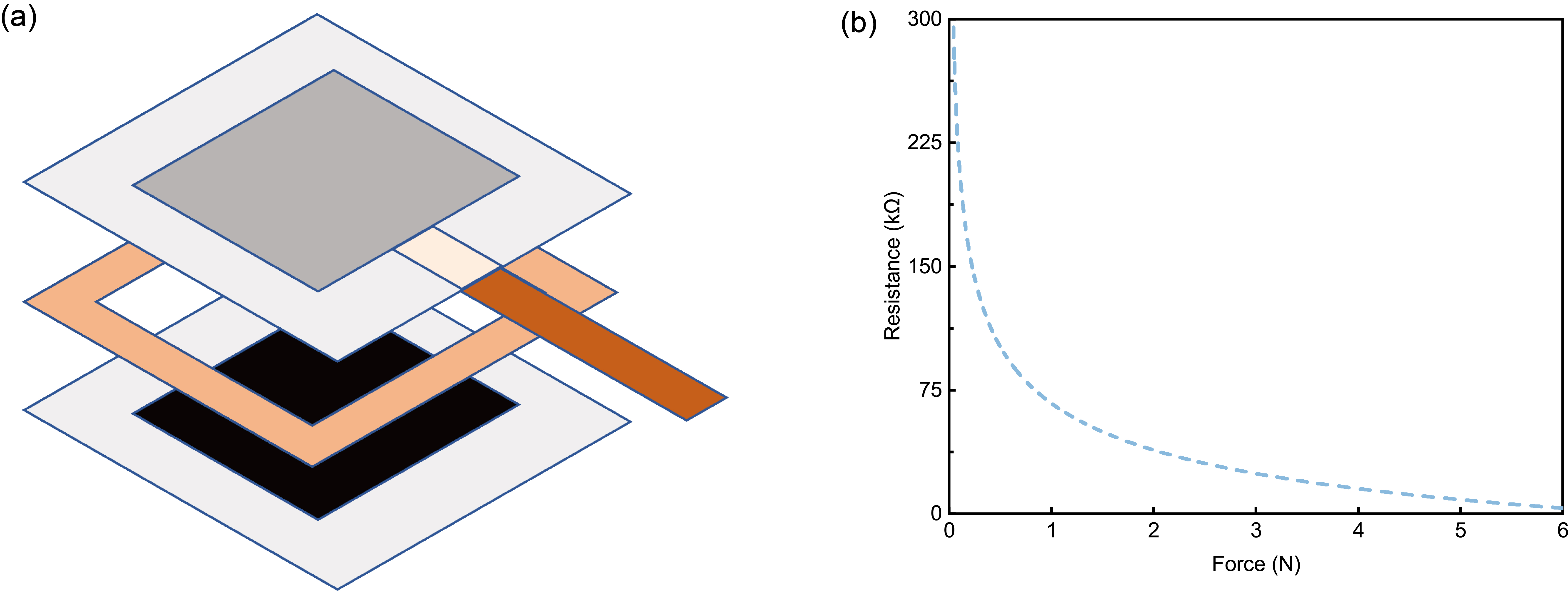


Fig S1. The structure and properties of the piezoresistive film. The specific structure of the device consists of the following layers: a protective layer of thermoplastic polyurethane elastomer rubber (TPU), a layer of conductive silver paste, a layer of pressure-sensitive material, and another layer of conductive silver paste, all enclosed between TPU protective layers. When external pressure is applied, the conductive silver paste makes contact with the pressure-sensitive material, enhancing conductivity and reducing the resistance of the device. Each point within the device can achieve a minimum resistance value of 10 kΩ, which increases to over 300 kΩ when no force is applied. In terms of performance, the piezoresistive film offers a wide range of force measurement capabilities, spanning from 0 to 6 N. The pressure characteristic curve exhibits a high degree of fitting. Specific fitting results can be observed in the figure.

## **Fig S2. The switching mechanisms of the self-directed channel (SDC) memristor.**


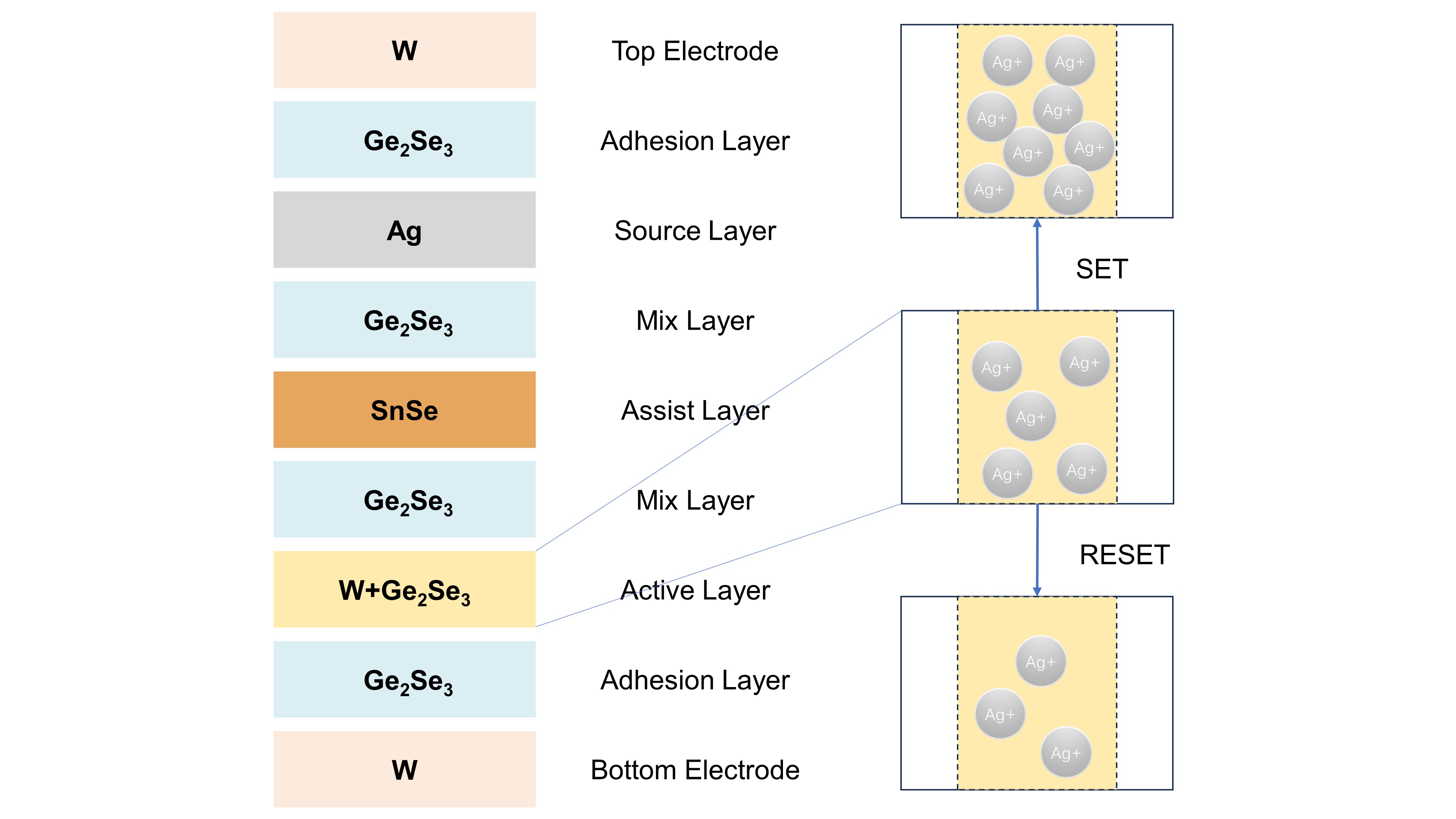


Fig S2. The switching mechanisms of the self-directed channel (SDC) memristor. Following fabrication, SDC devices start in a high-resistance state ranging from MΩ–GΩ. Upon first use of the device, the positive voltage applied to the top electrode can trigger the creation of the self-directed conductive channel in the SDC device. During this initial operation, Sn ions are generated from the SnSe layer and incorporated into the active Ge_2_Se_3_ layer. Theory suggests that these Sn ions promote the replacement of Ge by Ag at Ge-Ge bond sites in the active layer.

After Sn ions from the SnSe layer are incorporated into the Ge_2_Se_3_ layer, pairs of self-trapped electrons are produced within the Ge_2_Se_3_ layer. This process facilitates the replacement of Ge by Ag at the Ge-Ge bonds, creating “openings” near these bonds. These open regions serve as conduits for Ag^+^ ions, allowing them to access the Ag-Ge sites and become natural "conductive channels" within the active layer during device operation.

The self-directed channel follows with the position of the initial Ge-Ge dimers in the glass, influenced by its inherent structure. Due to the tendency of Ag atoms to agglomerate, these specific sites can foster Ag accumulation within the glass. This accumulation results in varied Ag concentrations at these clustering points. Consequently, the device's resistance is primarily decided by the Ag concentration at a given site and the distance between agglomeration sites. By applying positive or negative potentials across the device, Ag can be moved onto or away from these agglomeration sites, allowing the resistance to be tuned in both lower and higher directions.

## **Fig S3. The control circuit for the memristor.**


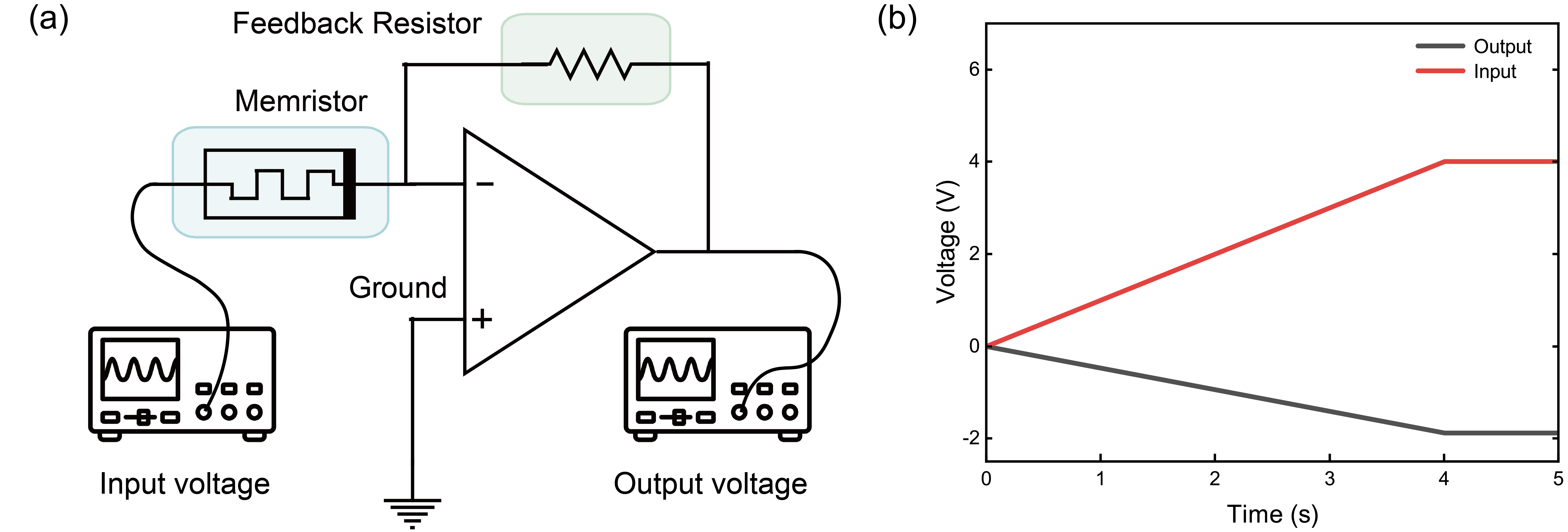


Fig S3. The control circuit for the memristor, based on the operational amplifier, is responsible for regulating the memristor state and providing feedback on its current state. The input terminal receives the encoded voltage from the signal generation module. By utilizing the negative feedback connection of the backend operational amplifier, the potential of the memristor bottom electrode is maintained at approximately 0 volts (virtual ground). This ensures that the voltage stimulus generated by the signal encoding remains undistorted and fully applies to both ends of the memristor, resulting in the modulation of the memristor. Moreover, the output voltage of the circuit allows for the feedback of the memristor state representing the long-term characteristics of the sensory stimuli.

## **Fig S4. The main system structure.**


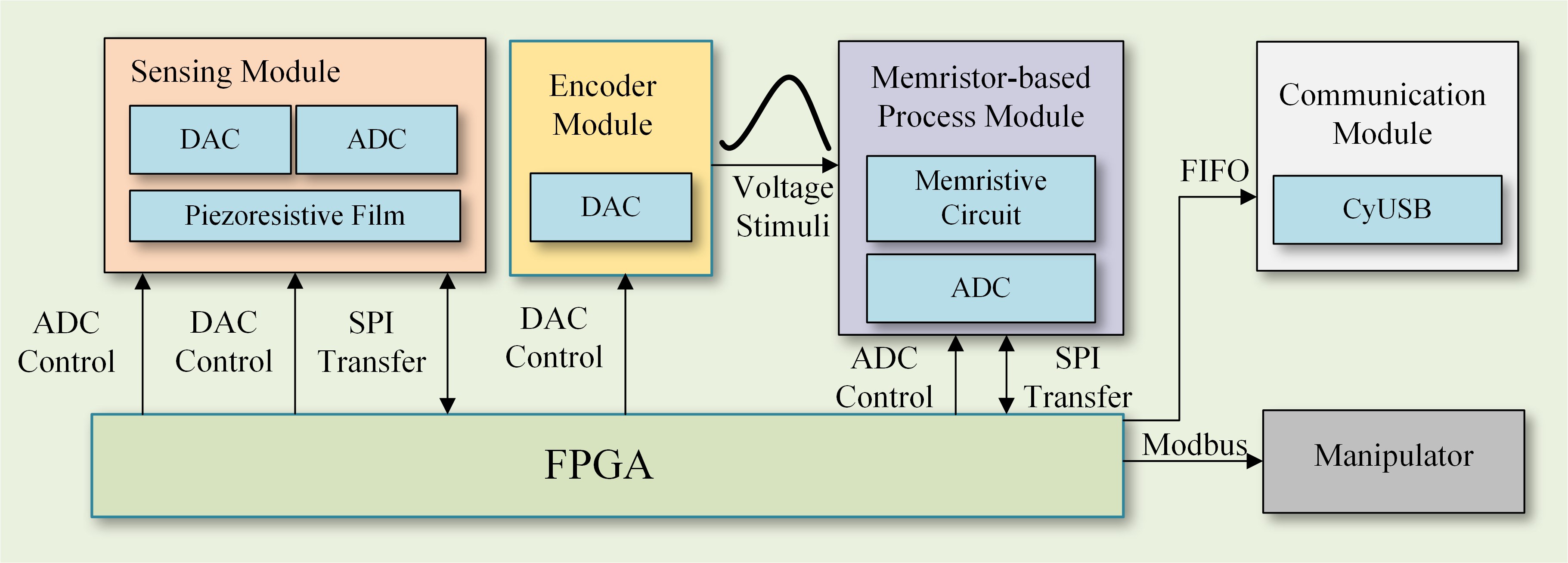


Fig S4. The main structure in tactile differential processing. The system is composed of several main components. The FPGA serves as the primary controller, with Intel's E4CE10F17C8 chosen as the FPGA main control chip. The external crystal oscillator operates at a frequency of 50 MHz. The front-end sensing module comprises a DAC, ADC, and piezoresistive film. The ADC, with the memristor process circuit, forms the memristor neuromorphic processing module. The DAC in the encoder module cooperates with the memristor-based process module to facilitate both the memristor resistance detection and the differential encoding generating. For communication, the CY7C68013A chip facilitates USB connectivity with the external host computer alongside CyUSB suites. This setup enables the transmission of current perception information and the state information of the memristor’s resistance value. In addition, the system features a communication interface designed to control the manipulator via the Modbus protocol, enhancing the capability of the robotic system to interact with external devices and environments.

## **Fig S5. The system background noise test.**


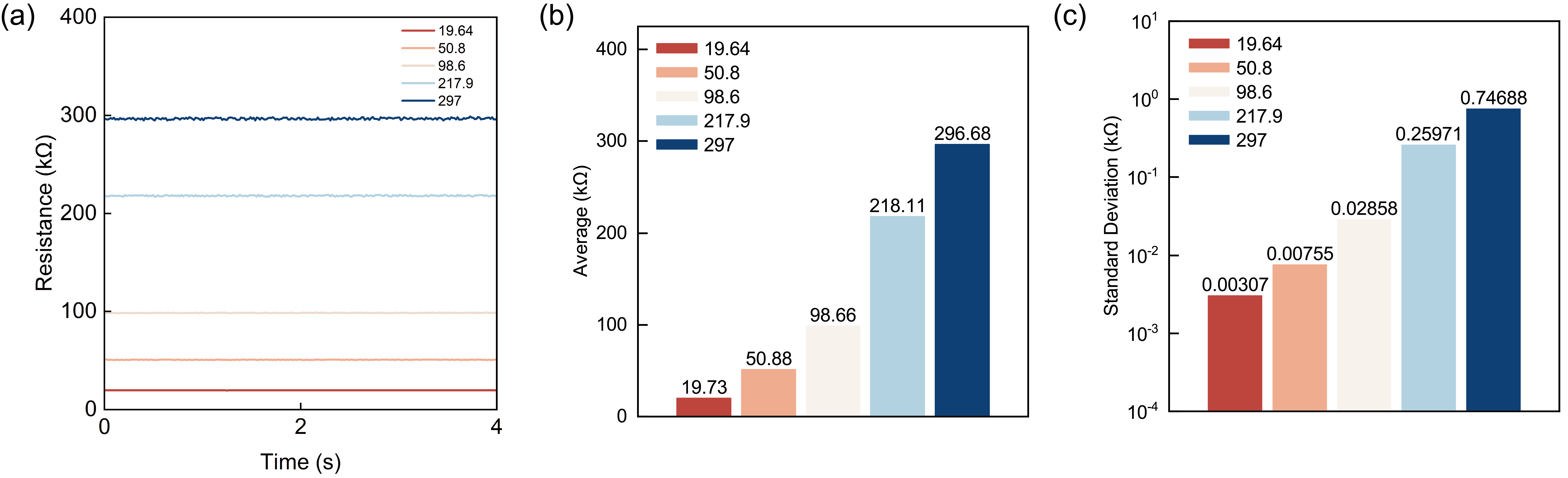


Fig S5. The system background noise test. (a) Measurement results of the fixed-value resistor. The memristor resistance device was substituted with a fixed-value resistor of varying values for testing purposes. By comparing the measured values with the actual values, it was observed that the overall system noise is relatively low. However, when testing a resistor with a high resistance value, it was found that the signal can be subject to fluctuations when exposed to noise. (b) The average value of the measured resistor. (c) The standard deviation of the measured resistor.

## **Fig S6. The different modulation types.**


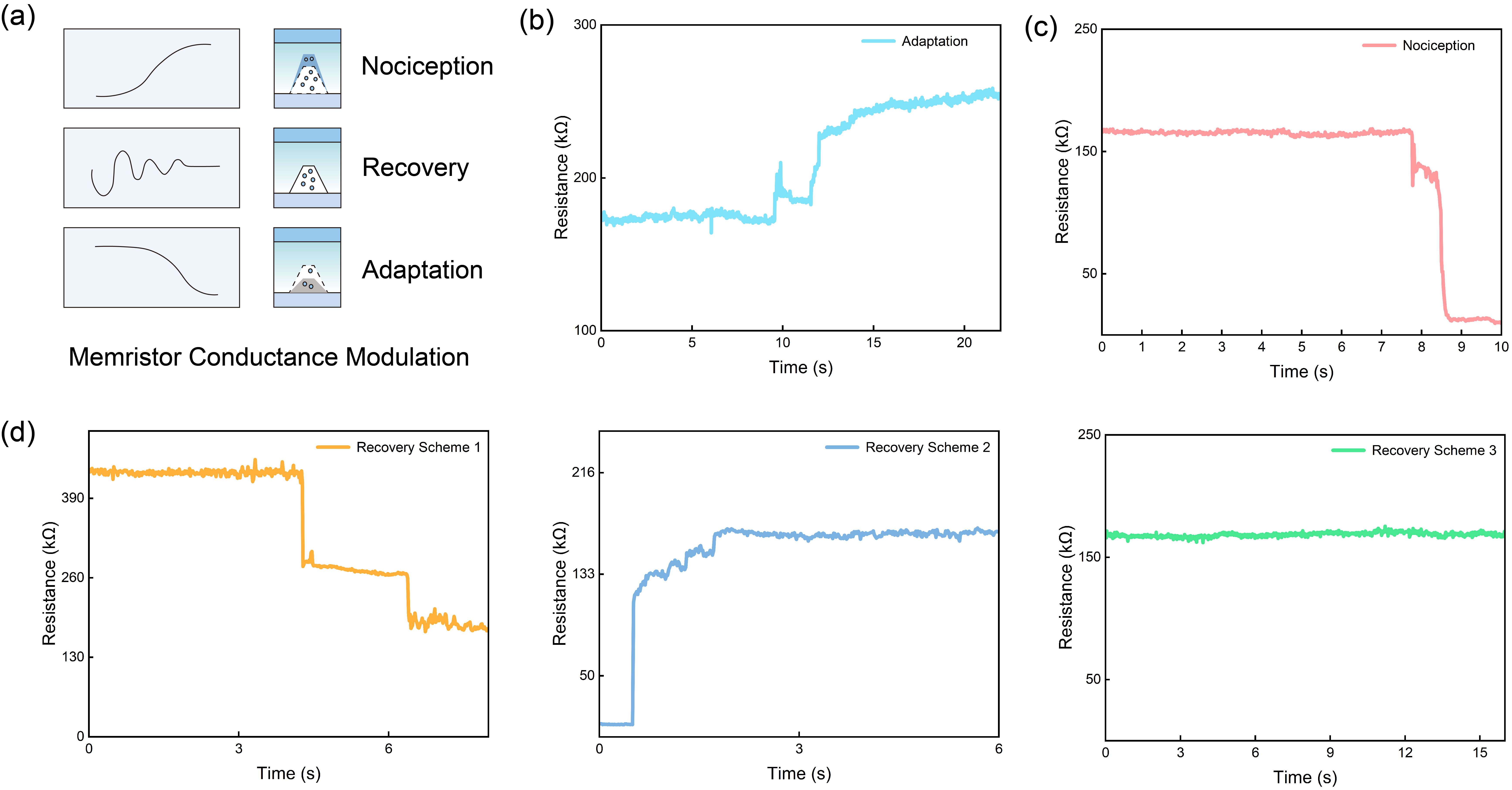


Fig S6. The memristor different modulation types. (a) In tactile information differential processing, there are three modulation modes: adaptation, nociception, and recovery. The amplitude of the modulation pulse of the adaptation type is negative, increasing the memristor resistance. In contrast, the amplitude of the nociception type is positive, decreasing the memristor resistance. The recovery pulse is responsible for resetting the memristor to the initial resistance interval; it is a negative pulse if the current state is low-resistance, a positive pulse if the current state is high-resistance, or no pulse is applied if it is already in the set interval. (b) The adaptation modulation pulse. (c) The nociception modulation pulse. (d) The recovery modulation pulse.

## **Fig S7. Tactile stimuli differential processing by biological receptors.**


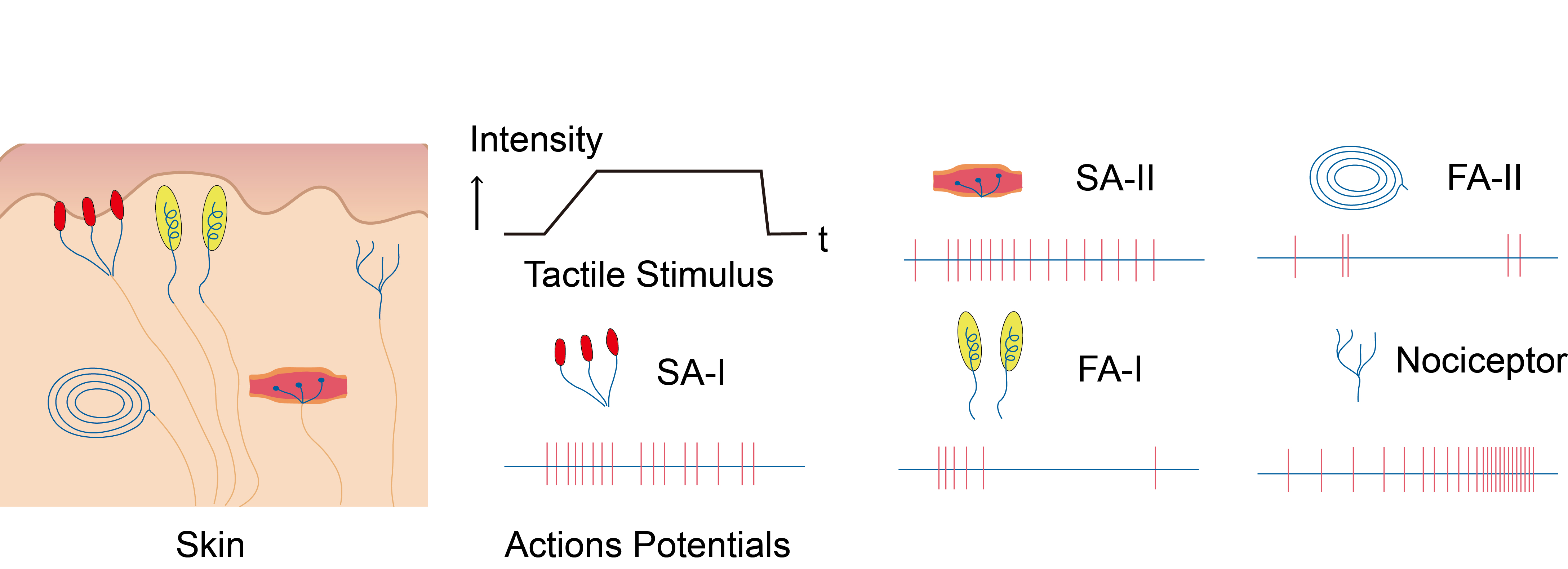


Fig S7. Differential processing of tactile stimuli by biological afferents. Two types, termed fast-adapting type Ⅰ (FA-Ⅰ) and fast-adapting type Ⅱ (FA-Ⅱ) afferents, respond only during dynamic phases of tissue deformation caused by tactile stimuli. Another two types, termed slowly adapting type Ⅰ (SA-Ⅰ) and slowly adapting type Ⅱ (SA-Ⅱ) afferents, respond to sustained skin deformation with graded sustained discharge. In addition, the nociceptor produces a sustained response to dangerous stimuli with increased intensity.

## **Fig S8. Other differential processing functions.**


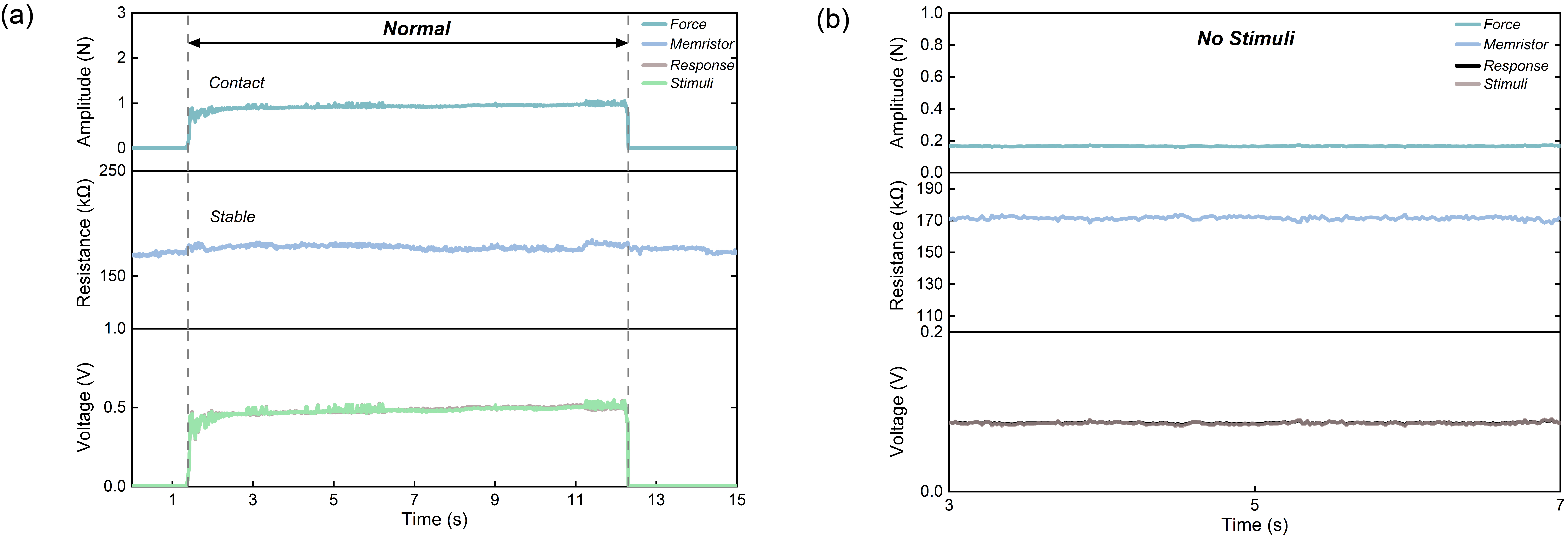


Fig S8. Other differential processing functions. During actual tactile perception, there are scenarios where there is no need to amplify or adapt to external tactile stimuli. For instance, when there is no external tactile stimulus present (as depicted in Figure S8 b), modulating the memristor would result in unnecessary energy consumption. Similarly, in another case, when the external pressure has a specific intensity but does not require amplification and the system is desired to accurately perceive its real intensity, there is no need to modulate the resistance value of the memristor. This corresponds to the normal process function depicted in Figure S8 a.

## **Fig S9. Processing functions in dynamic scenarios.**


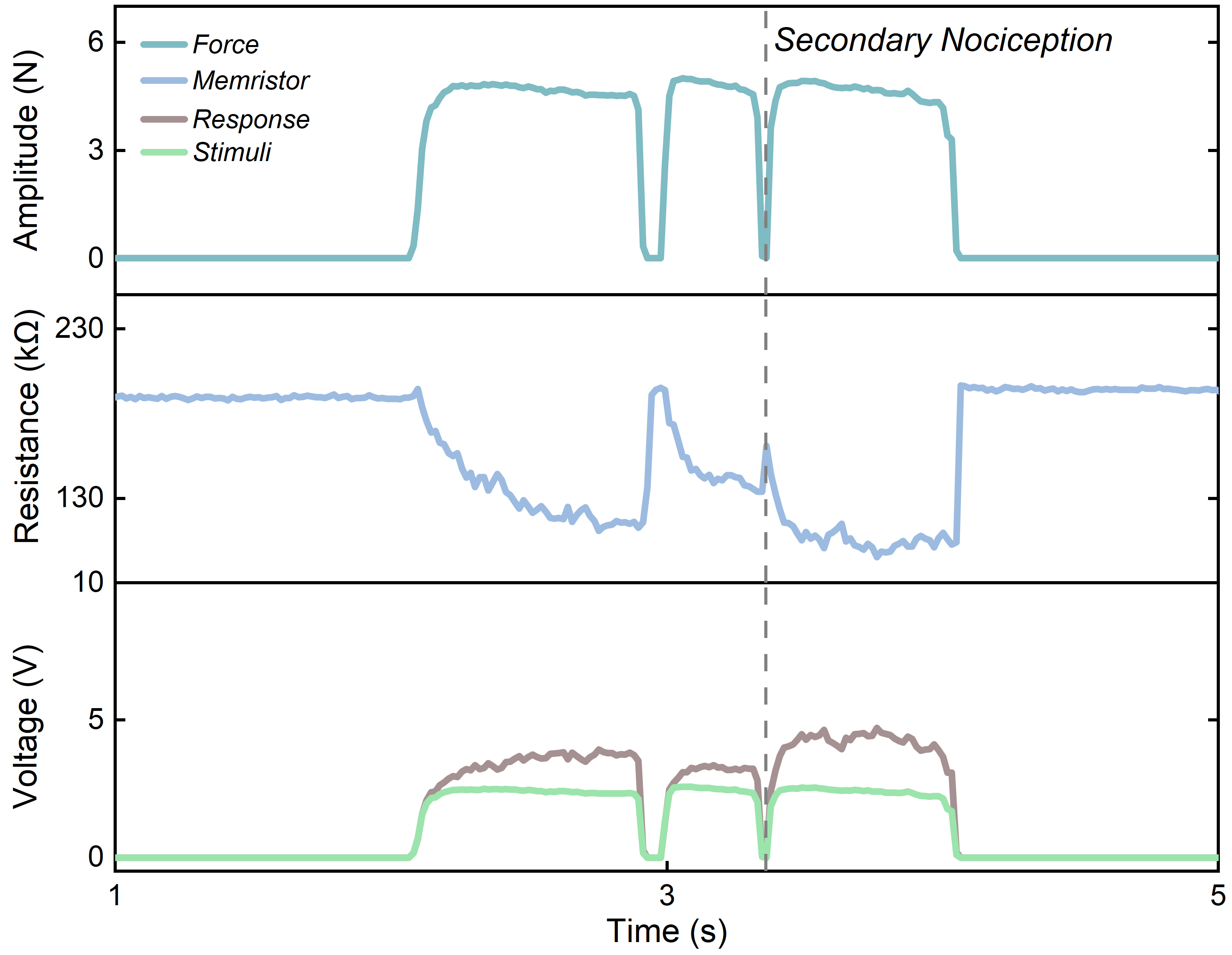


Fig S9. Processing functions in dynamic scenarios. In some dynamic scenarios, the features of the environmental information may change, and our proposed method is also capable of handling such dynamic information. As shown in Figure S9, when a dangerous stimulus is quickly withdrawn and then quickly applied again, the memristor resistance value has not yet reset completely. Thus, the memristor can achieve faster amplification of the external stimulus than that of the first stimulus, which demonstrates the function of ‘secondary nociception’.

## **Fig S10. Control logic for pain reflex and slip detection.**


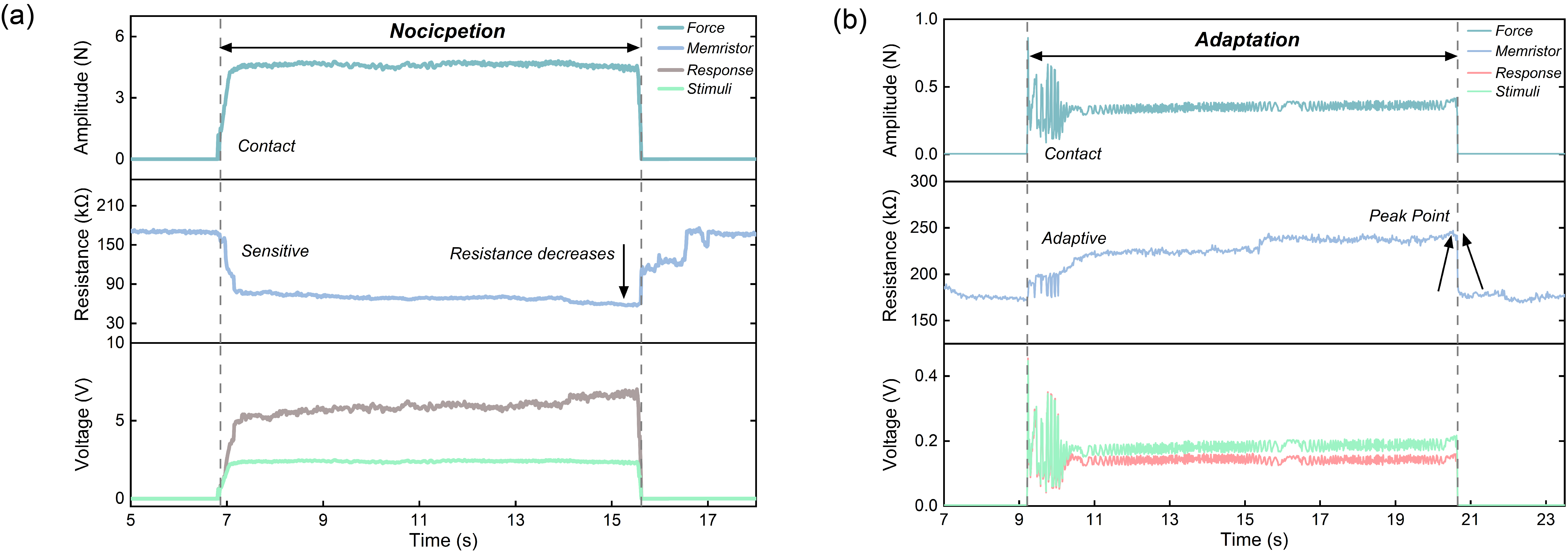


Fig S10. Control logic for pain reflex and slip detection. (a) The control logic for pain reflex. When working in unknown environments, robots need to have the ability to detect dangerous stimuli and provide a timely response. In such situations, the memristor is modulated to the low-resistance state under the positive pulses generated by the encoding module. When the memristor is less than a set threshold, it can be assumed that the robot has been subjected to dangerous stimuli for a period of time, and the pain reflex can be executed to protect the robot itself. (b) The control logic for slip detection. Once the robot has grasped the object to achieve stabilization, the slipping of the object primarily results from external interference. Thus, the memristor resistance will increase at first and decrease at the moment the object falls. The peak point of the memristor resistance will indicate the slipping of the object.

## **Fig S11. Memristor model used in visual information processing.**


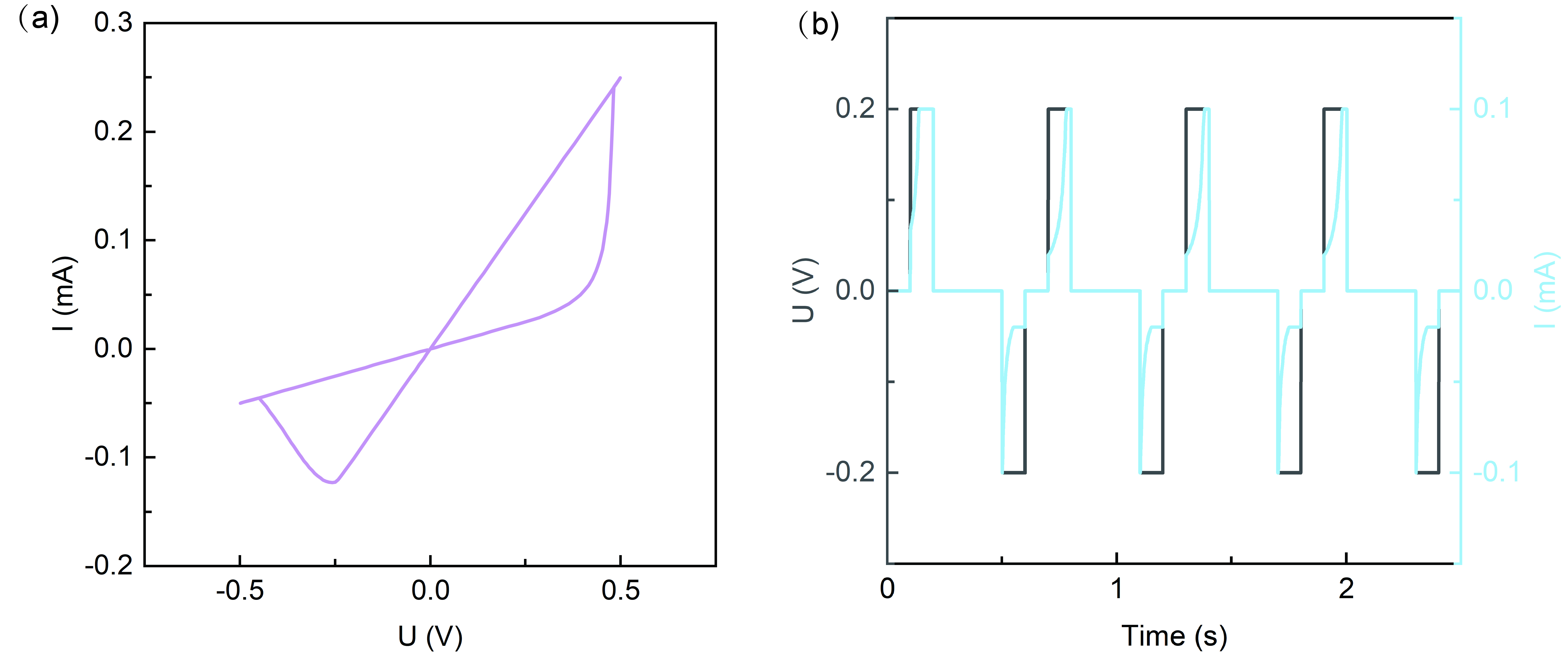


Fig S11. The voltage threshold adaptive memristor (VTEAM) model used in visual information processing. (a) The hysteresis curve of the memristor model. In this simulation, the tested sine wave has a peak-to-peak value of 1 V, and its frequency is 20 Hz. (b) The pulse test result of the memristor model. In this simulation, the amplitude of the pulse is 0.2 V, with a 0.1 s duration.

## **Fig S12. Noise Testing of Vision Circuits.**


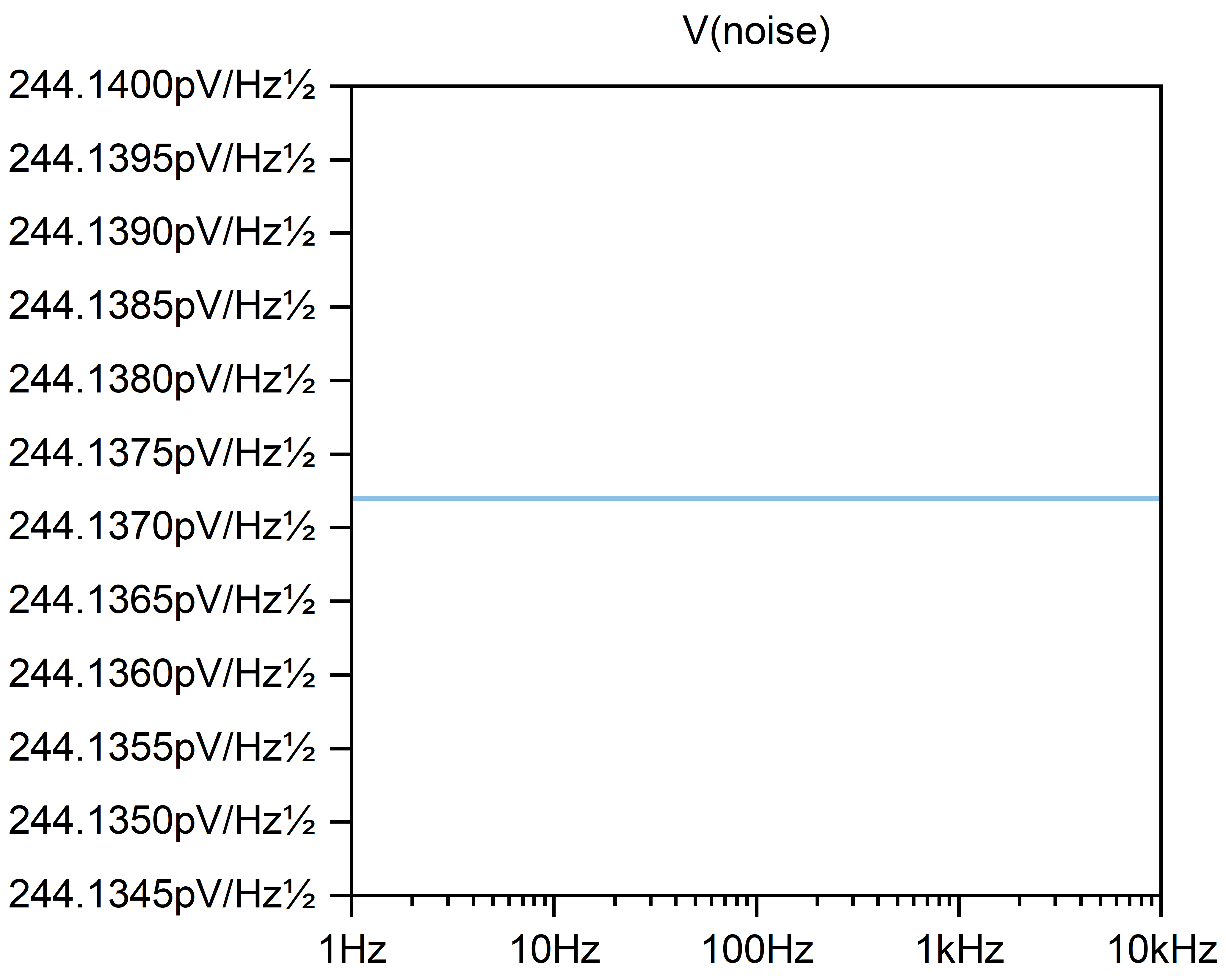


Fig S12. Noise Testing of Vision Circuits. To assess the noise levels in the vision circuits, we conducted a noise test. Assuming the external analog visual signal input is noise-free, we calculated the total noise that accumulates in the input voltage applied to the memristor read-write circuit. Through our calculations, we determined that the circuit noise level is approximately 200 pV.

## **Fig S13. Influence of threshold selection in visual differential processing.**


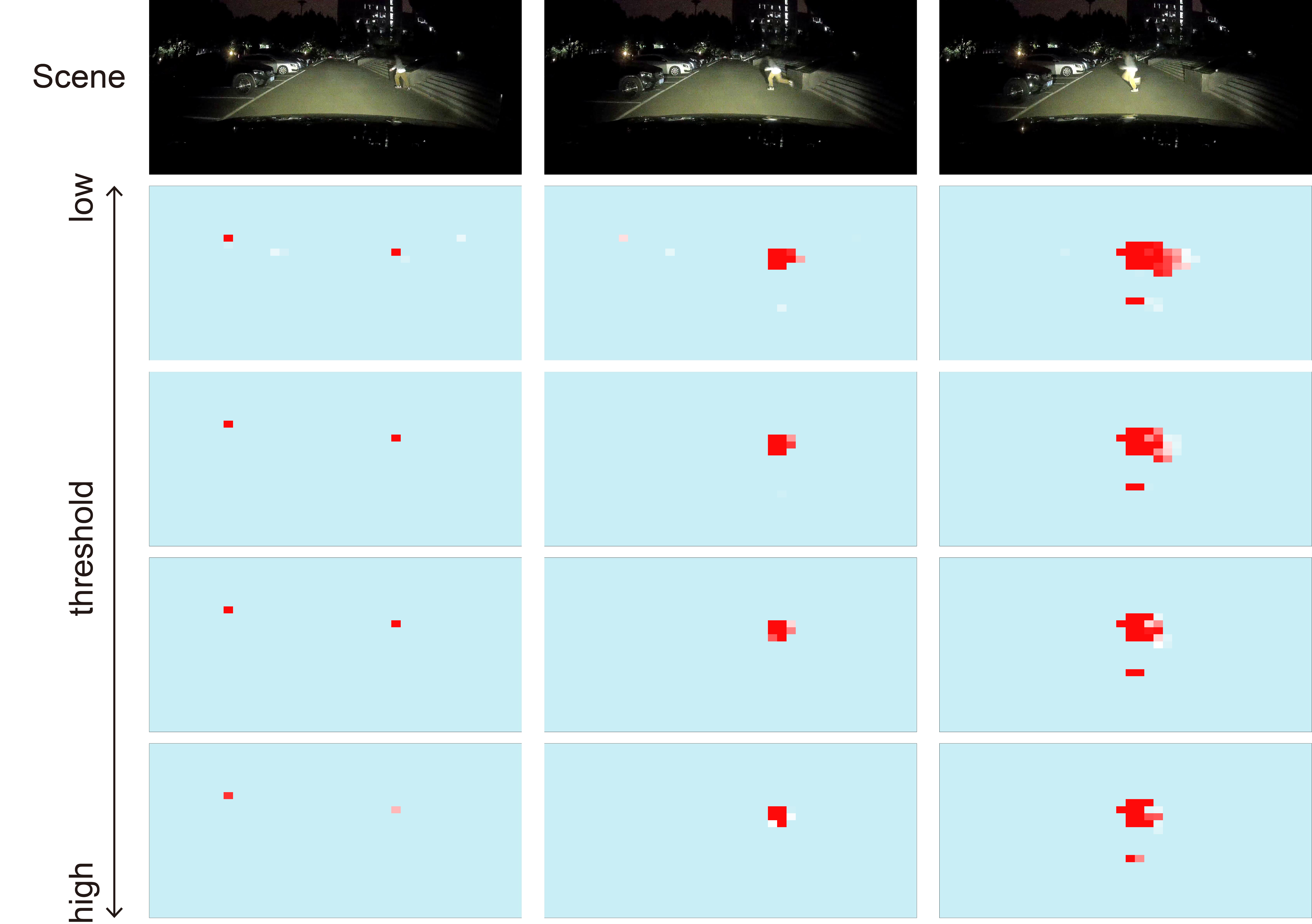


Fig S13. Influence of threshold selection in visual differential processing. In visual differential processing, the choice of threshold is pivotal in defining the properties of visual information. A positive voltage modulation scheme is generated only when the change frequency surpasses the threshold, leading the memristor into a low-resistance state. This signifies the presence of crucial object information during driving scenarios. As depicted in the accompanying figure, a higher selected threshold results in a smaller extracted area. However, the extracted information becomes more critical and pertinent. Concurrently, the duration of neural excitation is reduced with a high selected threshold. This is because a larger recovery negative voltage is more readily generated in this context.

## **Fig S14. Impact of object distance on system detection performance.**


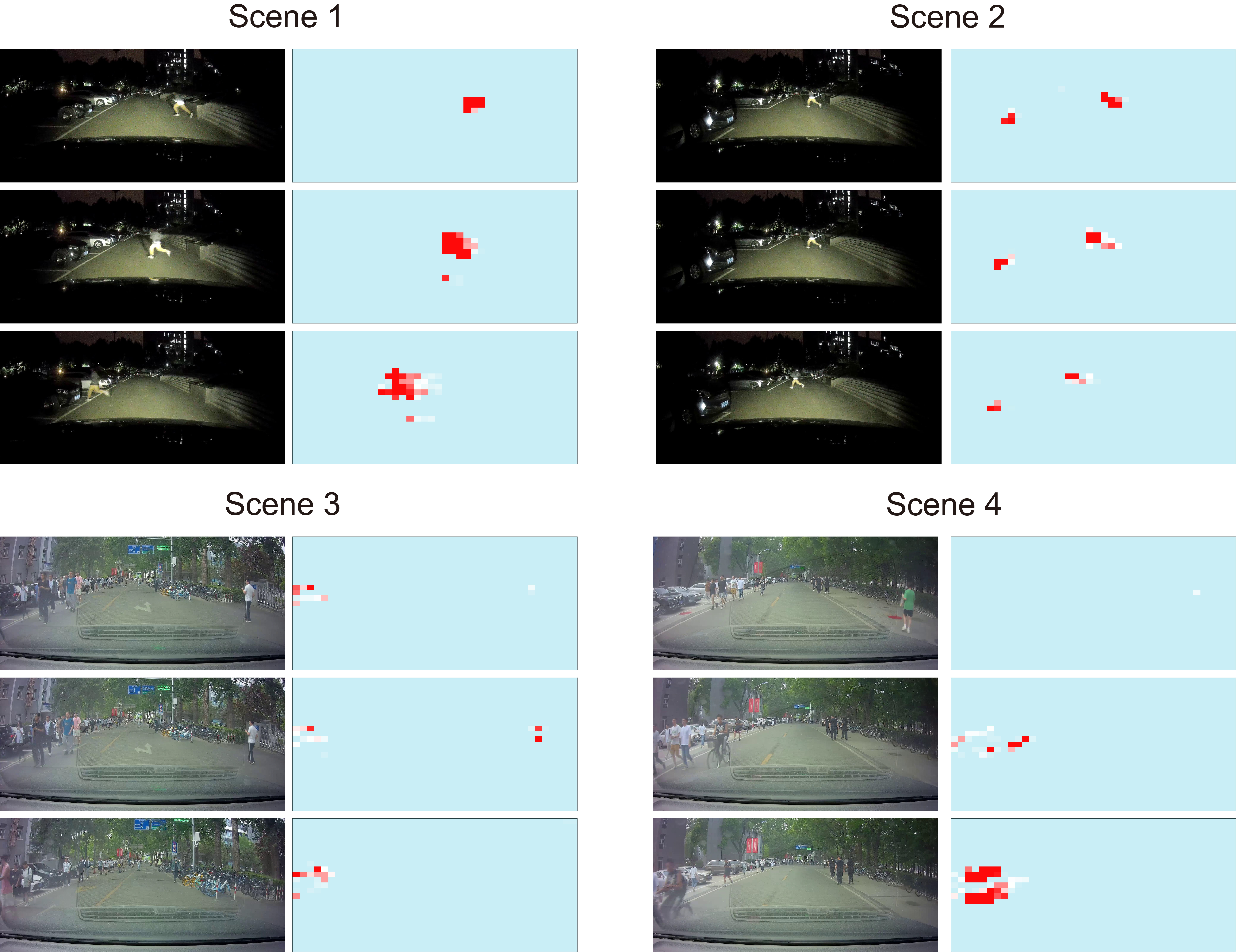


Fig S14. Impact of object distance on detection performance. The distance of objects within a compressed image influences their apparent size. Specifically, objects that are closer to the driver occupy a larger area in the image. As a result, nearby objects yield a larger extracted area than those that are distant, as demonstrated in Scene 1 and Scene 2. Moreover, an object moving at a constant speed becomes more detectable when it is closer, as illustrated by the comparison between Scene 3 and Scene 4.

## **Fig S15. Detection of road markings.**


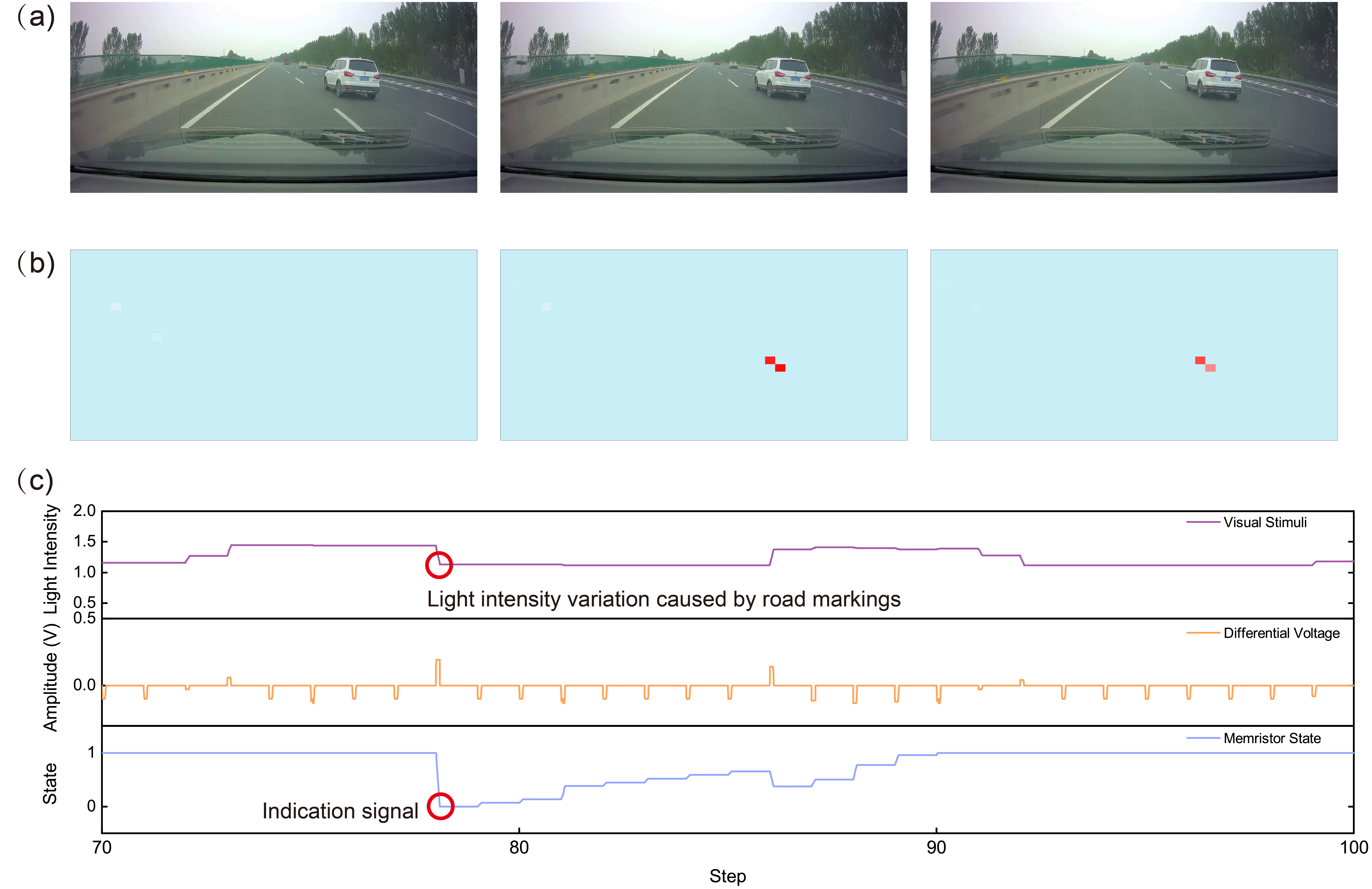


Fig S15. Detection of road markings. (a) Actual driving scenarios in the detection of road markings. While the road marking line itself remains stationary, the motion of a car induces relative movement, leading to variations in light intensity. This phenomenon enables the detection of the road marking line. (b) The detection results. (c) The light intensity change and its corresponding differential voltage and memristor state.

## **Fig S16. Detection of light sources.**


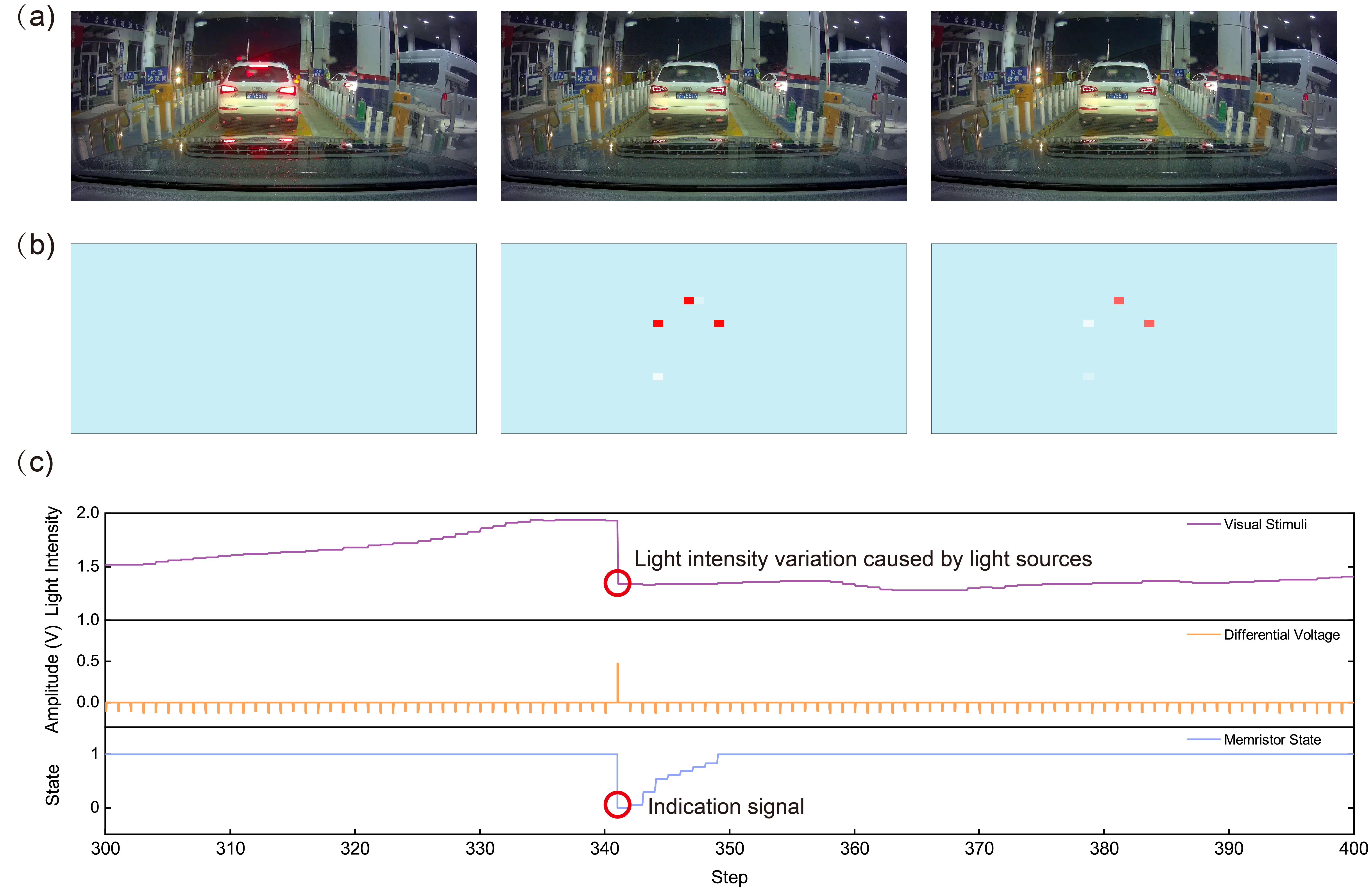


Fig S16. Detection of light sources. (a) Example scenario in the detection of light sources. When the tail lights of a car in the foreground abruptly illuminate or extinguish, a high-frequency change in light intensity ensues. This induces modulation of the memristor into a low-resistance state. (b) The detection results. (c) The light intensity change and its corresponding differential voltage and memristor state.

## **Fig S17. Analysis of detection performance for moving individuals.**


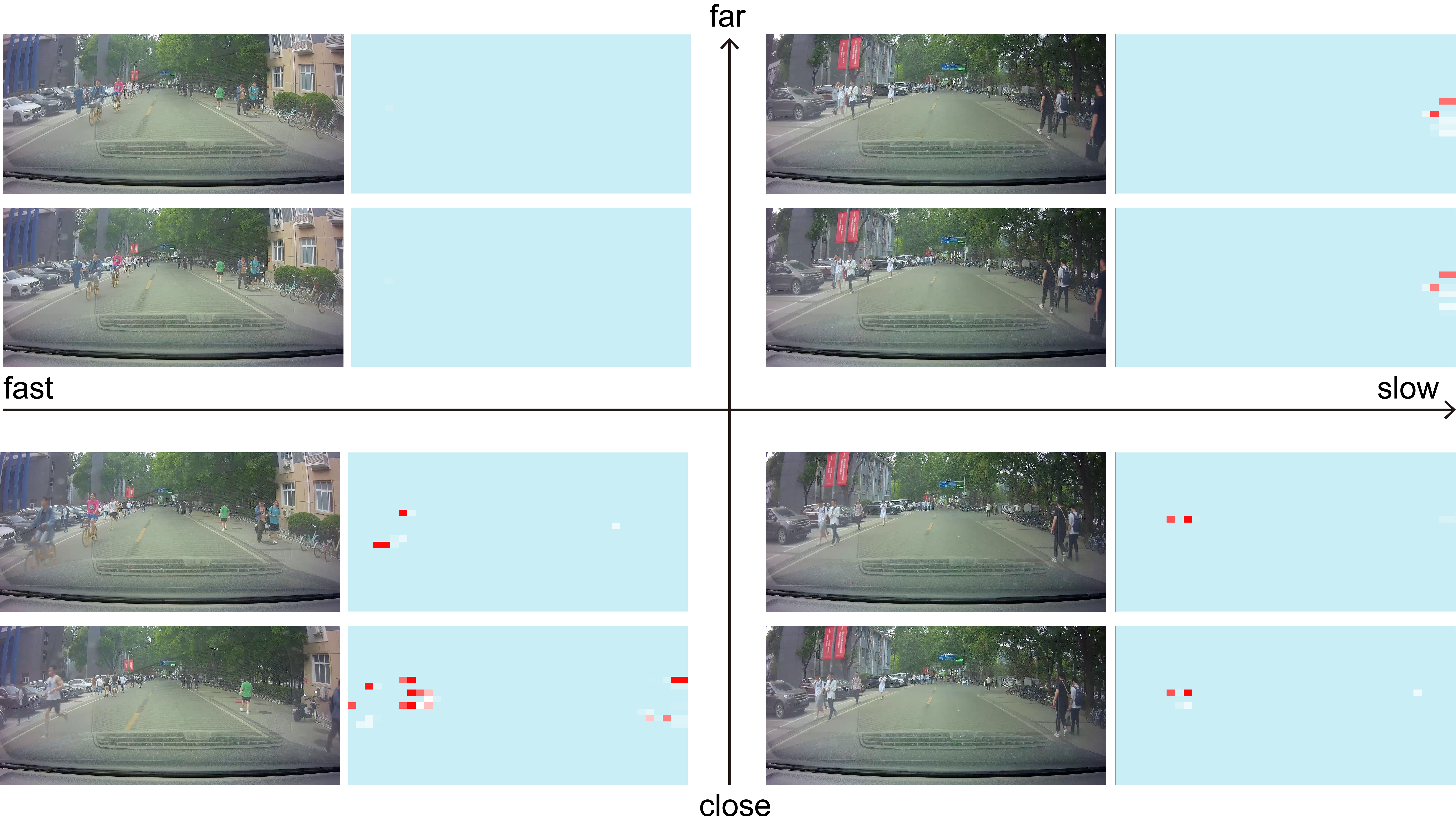


Fig S17. Analysis of detection performance for moving individuals. In the detection of a moving pedestrian, the performance is mainly affected by the position and speed of the pedestrian. Pedestrians are more likely to be detected when they are in close proximity and moving fast.

## **Fig S18. Detection for extremely hazardous scenarios.**


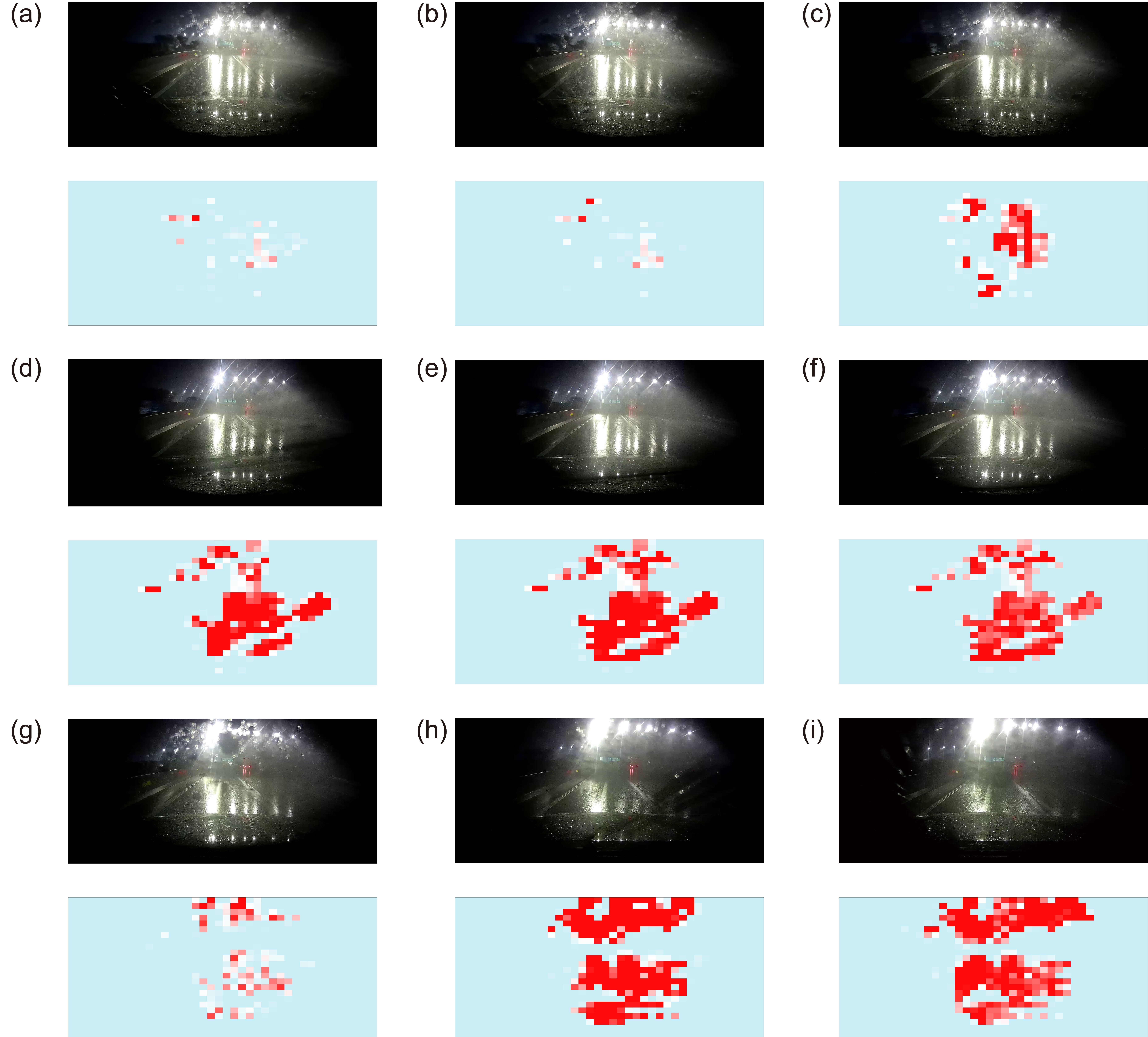


Fig S18. Detection for extremely hazardous scenarios. In certain driving scenarios characterized by abrupt fluctuations in ambient light intensity, depicted in (a) through (i), a significant majority of memristors in the detection results exhibit a low-resistance state. This phenomenon of pervasive low-resistance states can be utilized to characterize challenging driving environments.

## **Fig S19. Detailed detection results in unstructured environments.**

Fig S19. The extended results of Figure 5. In these extended results, more moments are used to reflect the changes in the scene as well as the detection results.

## **Fig S20. Processing of temperature sensing information.**


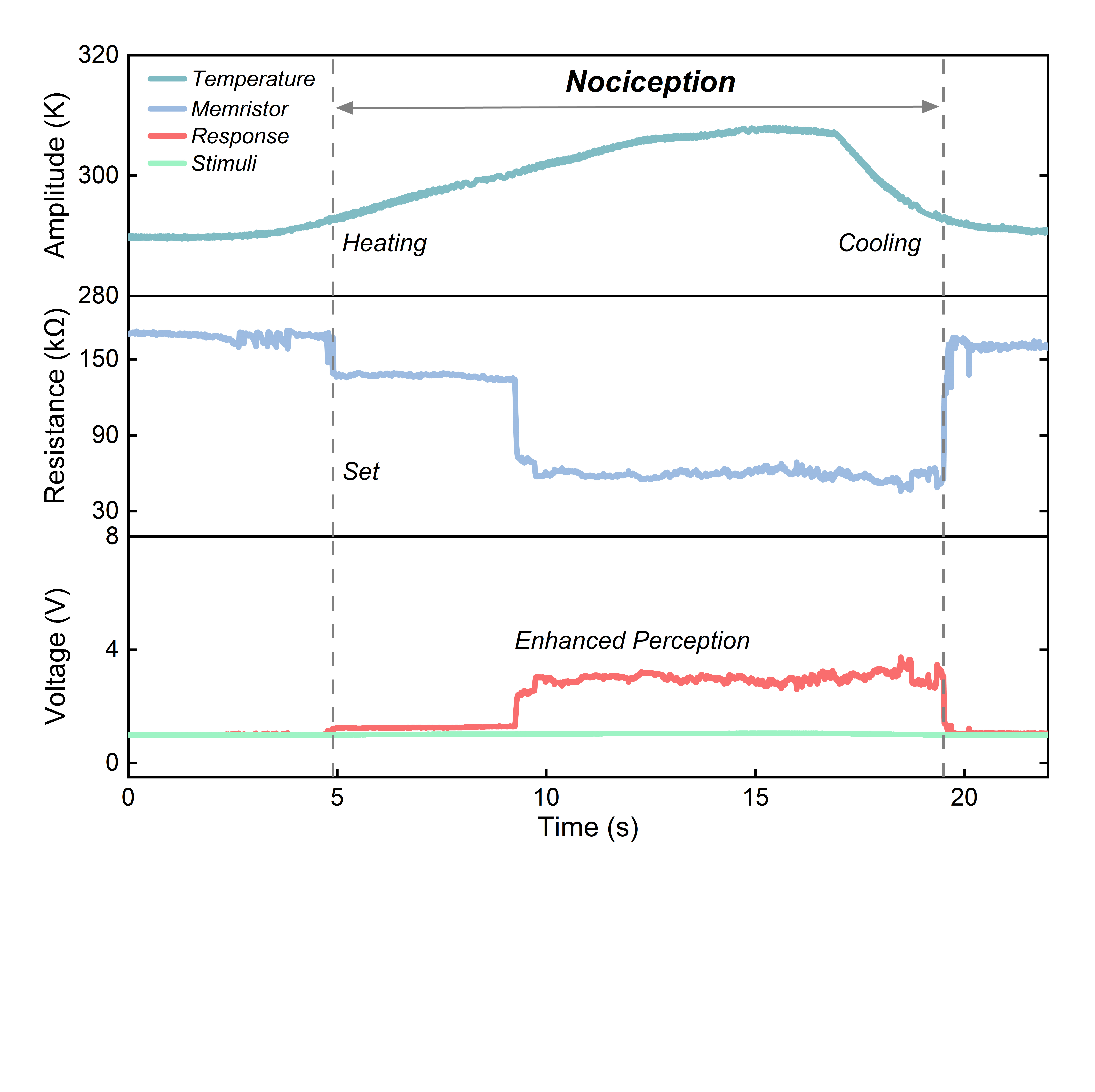


Fig S20. Processing of temperature sensing information. When the environmental temperature increases to exceed the set threshold, the memristor enters the set state with decreasing resistance, producing enhanced perception.

## **Fig S21. Processing of humidity sensing information.**


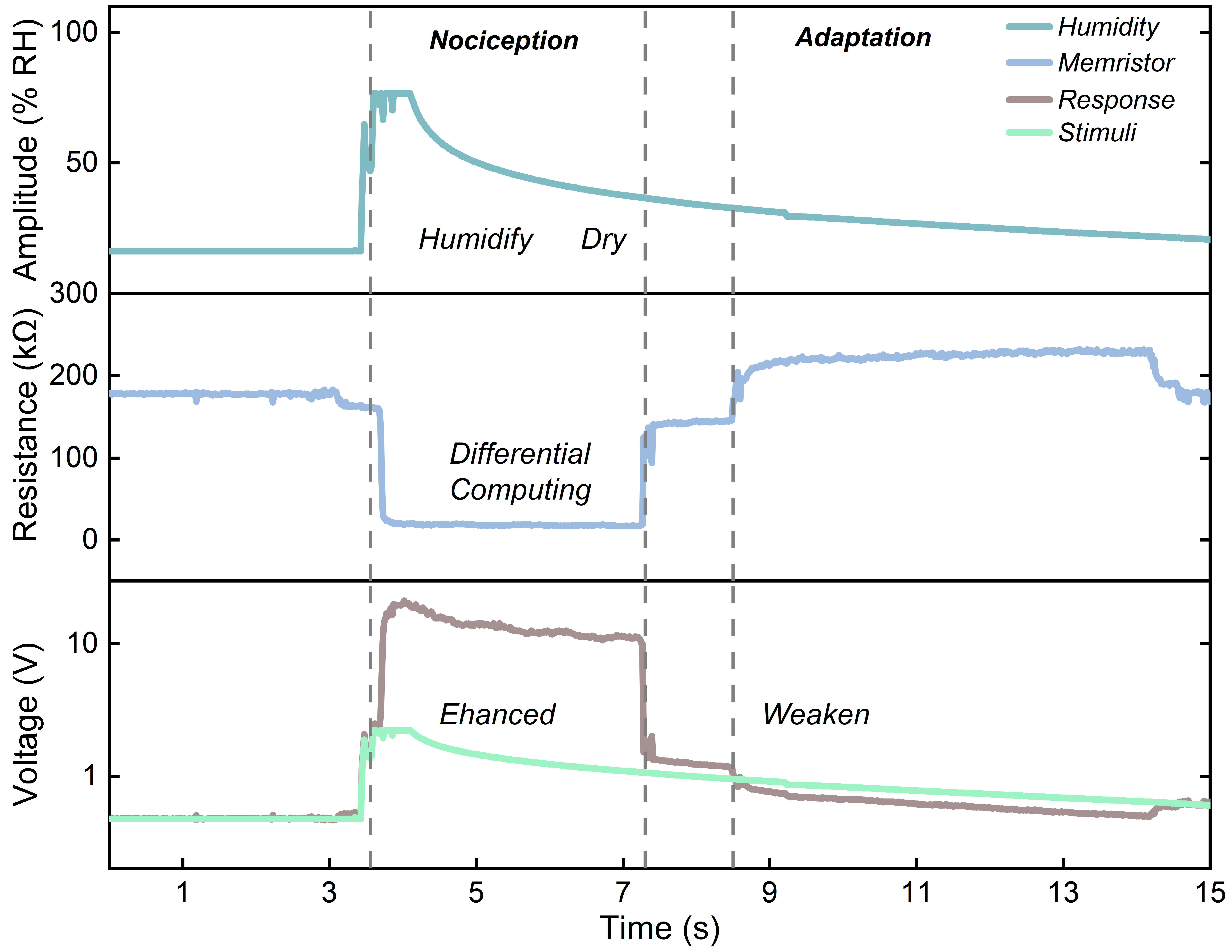


Fig S21. Processing of humidity sensing information. During this experiment, humidity experienced gradual increments and decrements, leading to modulation schemes of nociception, recovery, adaptation, and recovery. Consequently, the memristor states transition sequentially from low-resistance to middle-resistance, then to high-resistance, and back to middle-resistance states. Thus, a single memristor can embody enhanced, adaptation, and normal perception functions through differential computing methods.

## **Fig S22. The differential processing model.**


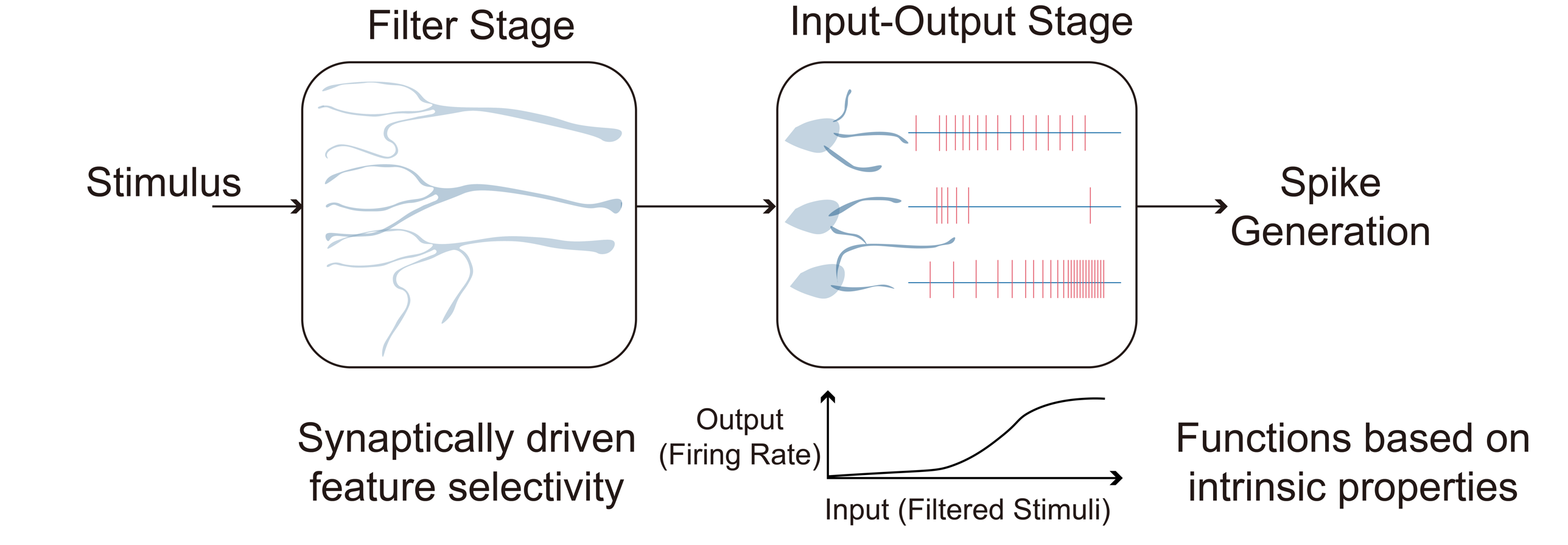


Fig S22. The differential processing model. From perceiving the stimulus to encoding it into neural impulses, the model can be classified into two stages. The first stage can be considered a filter stage where the sensory stimulus is filtered by the feature selectivity of neurons. The second stage can be described as an input-output stage that transforms the filtered stimulus input into a firing rate output. Ultimately, the neural pathways will process these spikes differentially.

**Fig S23.** **Comparison between other works.**


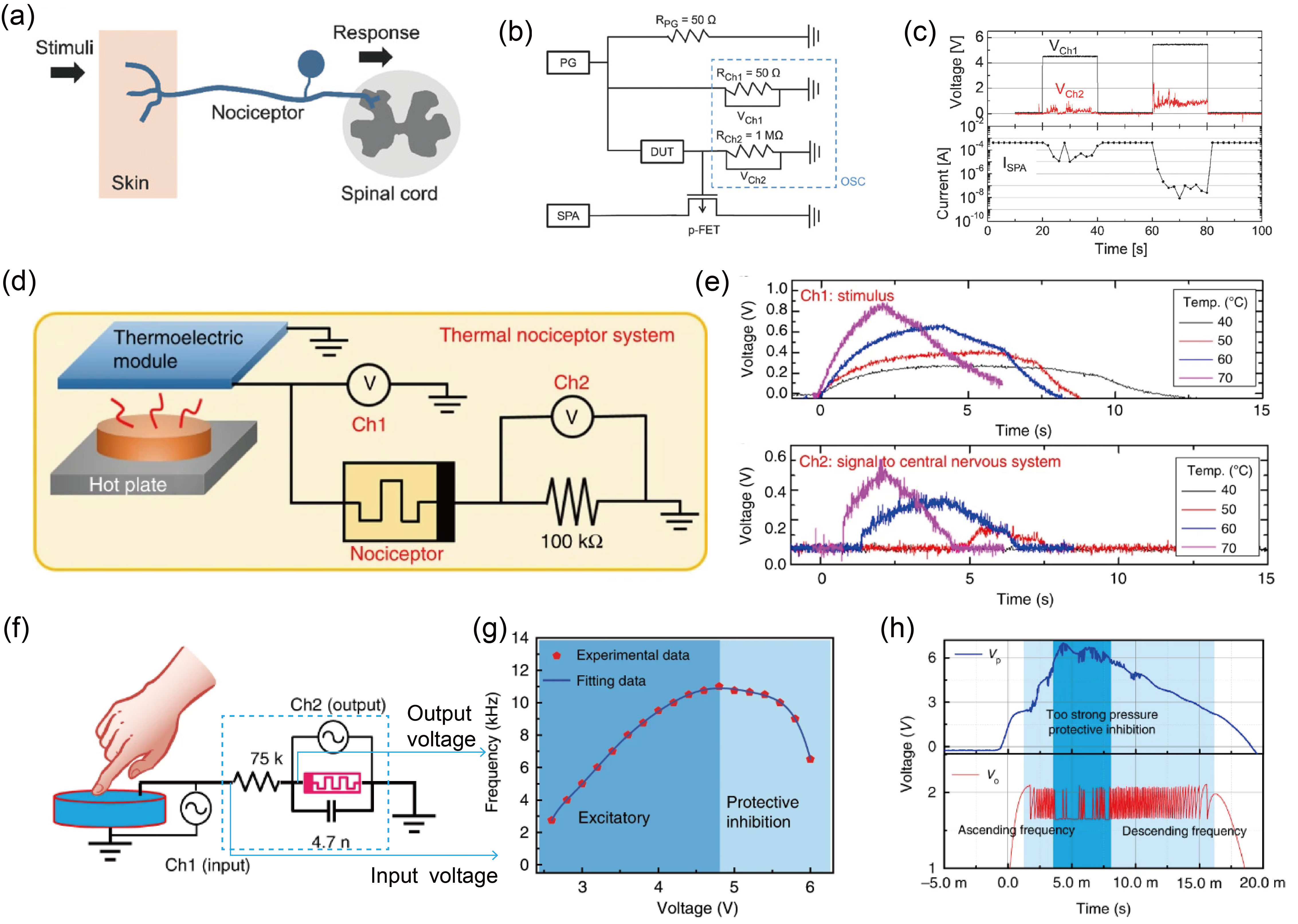


Fig S23. Comparison between other works. (a) Schematic diagram of the typical nociceptor nervous system. (b) The circuit configuration of a memristor-based artificial nociceptor. Note that the DUT (device under test) represents the memristor. (c) Input voltages (*V*_Ch1_) produced by the pulse generator, and output voltages (*V*_Ch2_) applied to the p-FET (upper panel), and currents (*I*_SPA_) measured by the semiconductor parameter analyzer (lower panel). a-c reproduced with permission (*23*). (d) Schematic diagram of an artificial thermal nociceptor consisting of a thermoelectric module and the diffusive memristor. (e) The generated voltage from the thermoelectric module and the ON-switching and OFF-switching of the threshold switch monitored by Ch1 and Ch2 of the oscilloscope, respectively. d-e reproduced with permission (*22*). (f) Schematic of the artificial mechanoreceptor system. In this system, a piezoelectric device acts as the tactile sensor for receiving external sensory information and is connected to the artificial afferent nerve. (g) The relationship between the input voltage and the frequency of response voltage of the artificial afferent nerve. (h) The frequency response of the system under external pressure. f-h reproduced with permission (*62*).

**Fig S24. The switch in modulation schemes achieved by FPGA controlling.**


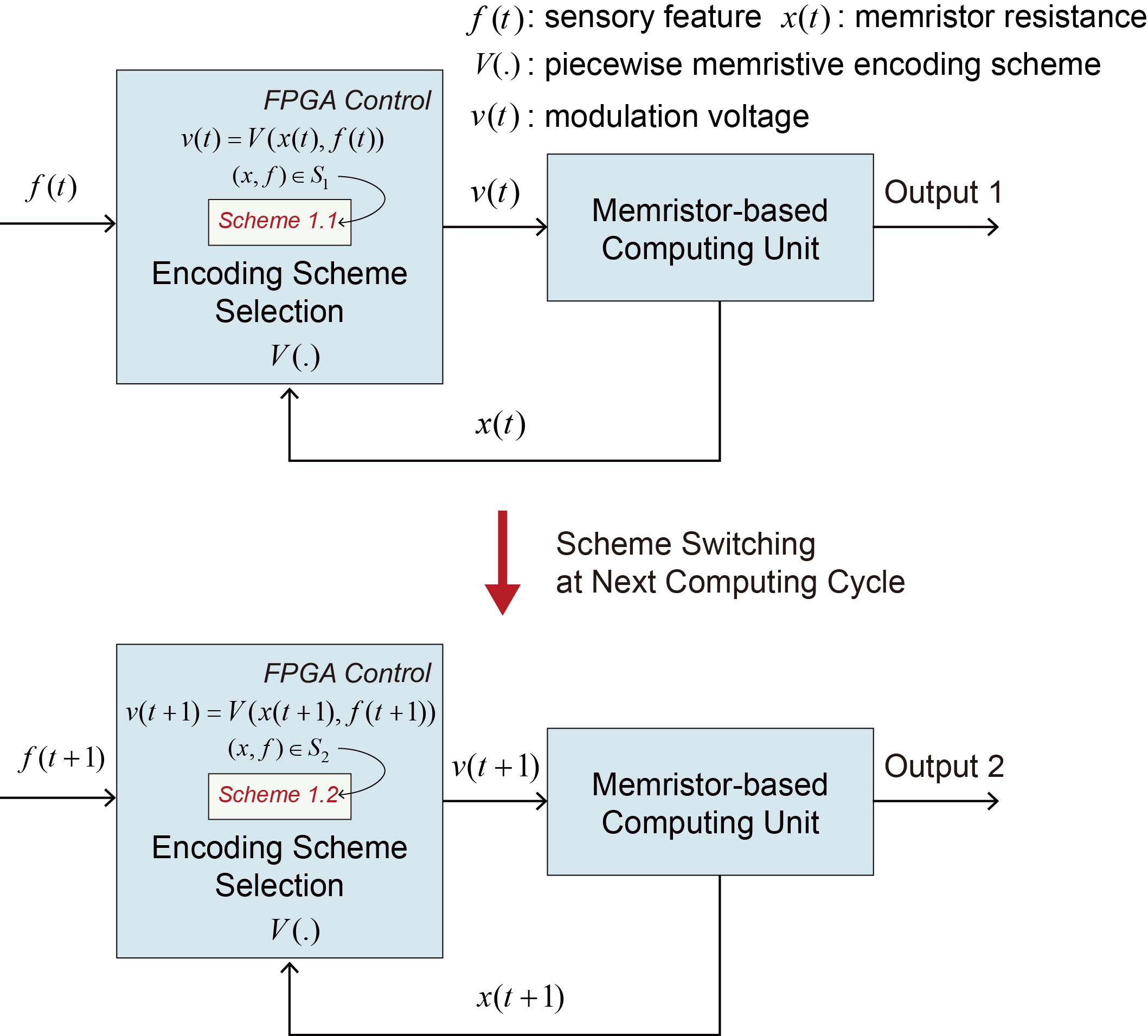


Fig S24. The switch in modulation schemes achieved by FPGA controlling. When the sensory stimuli feature is in a constant hazardous condition, the system selects the encoding strategy that adjusts the memristor to a low-resistance state. This strategy bifurcates into two distinct modulation schemes based on the memristor resistance : Scheme 1.1, which operates at a 13.3% duty cycle and a 0.30 V voltage amplitude, and Scheme 1.2, featuring a 20% duty cycle and a 0.45 V voltage amplitude. Scheme 1.1 is selected when the pair $(x(t),f(t))$ falls within Set $S_{1}$, i.e., the memristor resistance exceeds the predefined threshold of 100 kΩ, and the feature is hazardous. If the memristor resistance at the next computing cycle is below this 100 kΩ threshold, indicating the pair $(x(t+1),f(t+1))$ belongs to Set $S_{2}$, the scheme switches into scheme 1.2, achieved by FPGA controlling.

**Fig S25. Comparison between the image captured by a standard car camera, the output from an event-based camera, and the outcomes achieved through the differential neuromorphic computing approach.**


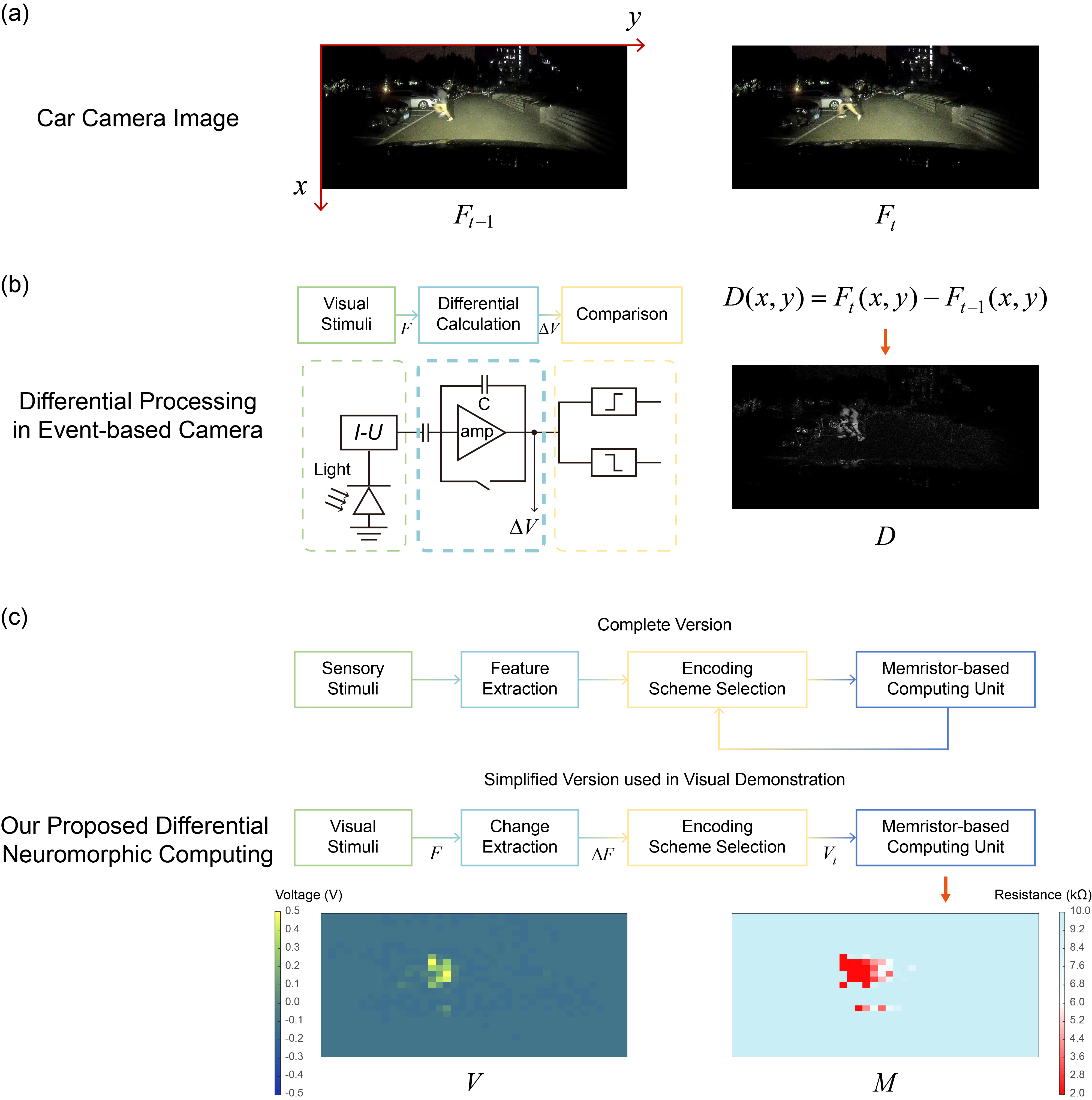


Fig S25. Comparison between the image captured by a standard car camera, the output from an event-based camera, and the outcomes achieved through the differential neuromorphic computing approach. In event-based cameras, a specialized circuit is designed to perform differential processing, i.e., detect the light intensity change, denoted as $\Delta V$. When $F_{t}$ represents the static image at time $t$, the light intensity change $\Delta V$ can be discerned by calculating $D$. In the visualizing results, it is observed that the differences between two consecutive frames are highlighted. Similarly, the differential encoding $V$ (similar to $D$) merely identifies changes between frames, and the information is noisy after compression. In contrast, the memristor array $M$ not only distinctly emphasizes rapid changes but also maintains historical change information, resulting in a clear position of the running pedestrian and the afterimages that imply his moving direction. These capabilities provide cleaner and more actionable data for further high-level processing, such as deducing the direction of movement through afterimages, predicting future locations, and other decision-making processes crucial for navigating dynamic environments. (a) Car camera image. (b) Differential processing in event-based camera. In event-based cameras, differential processing typically refers to the specialized circuit designed for detecting changes in light intensity, with the outcomes represented as $D$. (c) Our proposed differential neuromorphic computing. In our proposed differential neuromorphic computing, differential encoding mainly refers to the appropriate encoding scheme based on current sensory features. For our visual processing demonstration, we identify changes in the light intensity as the primary sensory feature, and the differential encoding pulses $V$ are applied to the memristor array, leading to the memristor states in $M$.

**Fig S26. Comparison between the differential neuromorphic computing and PID control.**


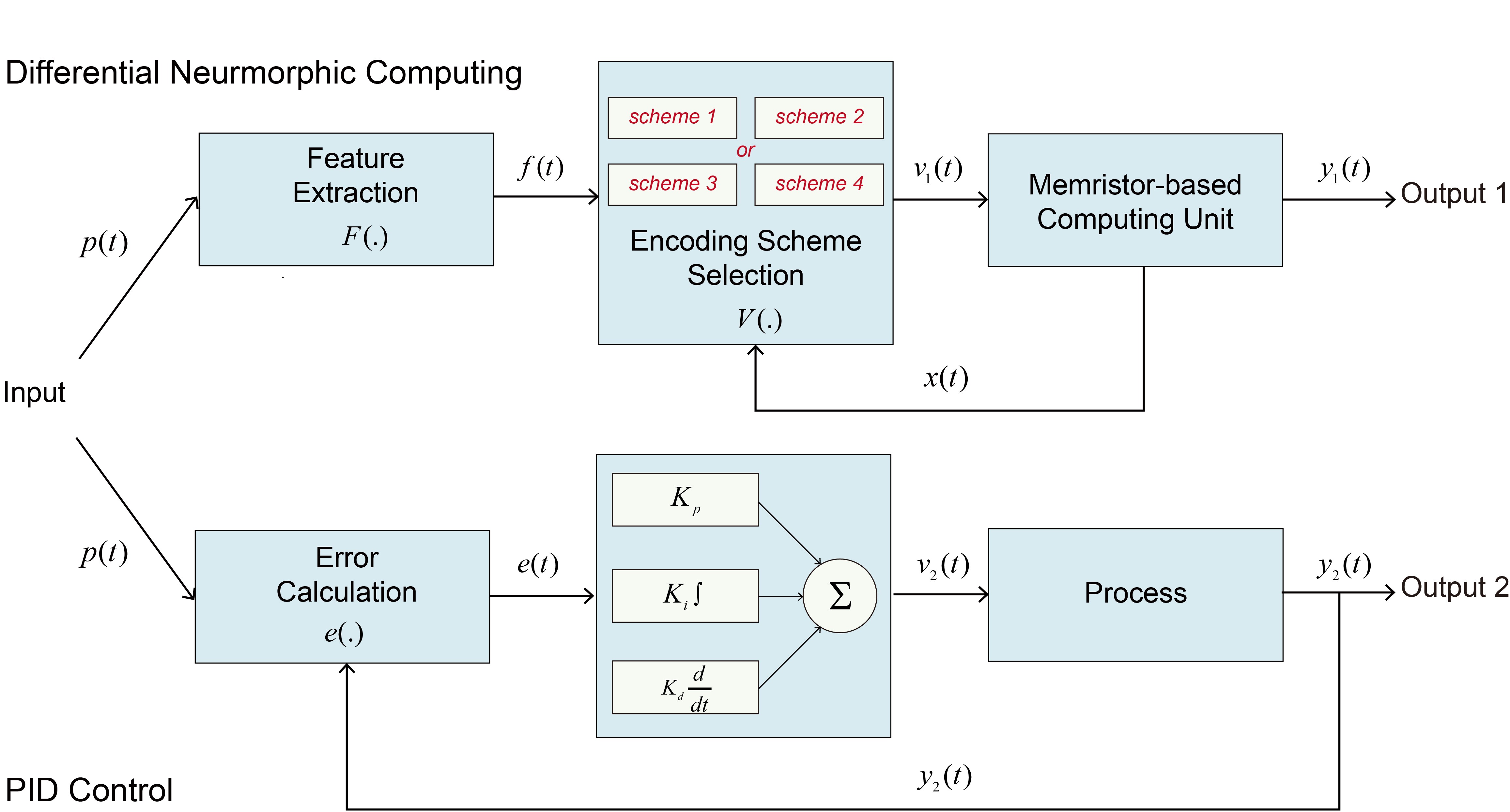


Fig S26. Comparison between the differential neuromorphic computing and PID control.

# Supplementary Tables

## **Supplementary Table 1 The modulation scheme**

| Stimulus Strength | Memristor state | Feature pattern | Encoding scheme |
| --- | --- | --- | --- |
| *R*_p_ < 60 kΩ | *R*_m_ > 100 kΩ | Noxious stimulus | 13.3% 0.30 V |
| *R*_p_ < 60 kΩ | *R*_m_ < 100 kΩ | Noxious stimulus | 20.0% 0.45 V |
| 60 kΩ *< R*_p_ < 100 kΩ | *R*_m_ > 250 kΩ | Normal stimulus | 26.7% 0.35 V |
| 60 kΩ *< R*_p_ < 100 kΩ | *R*_m_ < 100 kΩ | Normal stimulus | 20.0% -0.40 V |
| 60 kΩ *< R*_p_ < 100 kΩ | 180 kΩ *<* *R*_m_ < 250 kΩ | Normal stimulus | 6.7% 0.42 V |
| 60 kΩ *< R*_p_ < 100 kΩ | 100 kΩ *<* *R*_m_ < 160 kΩ | Normal stimulus | 6.7% -0.50 V |
| 100 kΩ *< R*_p_ < 300 kΩ | *R*_m_ > 100 kΩ | Mild stimulus | 46.7% -0.25 V |
| 100 kΩ *< R*_p_ < 300 kΩ | *R*_m_ < 100 kΩ | Mild stimulus | 6.7% -0.50 V |
| *R*_p_ > 300 Ko | *R*_m_ > 250 kΩ | No stimulus | 26.7% 0.35 V |
| *R*_p_ > 300 kΩ | *R*_m_ < 100 kΩ | No stimulus | 20.0% -0.40 V |
| *R*_p_ > 300 kΩ | 180 kΩ *<* *R*_m_ < 250 kΩ | No stimulus | 6.7% 0.42 V |
| *R*_p_ > 300 kΩ | 100 kΩ *<* *R*_m_ < 160 kΩ | No stimulus | 6.7% -0.50 V |

Note: The threshold selection in our modulation schemes is the result of careful hand-tuning based on the electrical characteristics of the memristor used as well as the modulation scheme. Taking the threshold 100 kΩ as an example, it is designed to recognize the condition when the system encounters dangerous stimuli for a while; the nociceptor enters into the sensitization state. In other words, the memristor has been modulated by the positive voltage pulses for a while. Thus, it is set below the memristor initial middle resistance value (~170 kΩ), and 100 kΩ effectively serves as an indicator of sustained exposure to dangerous stimuli.

## **Supplementary Table 2 Comparative Analysis of Visual Information Extraction Algorithm Complexities**

| Algorithms | Time Complexity | Space Complexity |
| --- | --- | --- |
| Gaussian Blur | *O*(*nmk^2^*) | *O*(*nm*) |
| Canny Edge Detection | *O*(*nm*) | *O*(*nm*) |
| Histogram Equalization | *O*(*nmk^2^*) | *O*(*nm*) |
| SIFT | *O*(*nm*) | *O*(*nm*) |
| SURF | *O*(*nm*) | *O*(*nm*) |
| Our Work | *O*(1) | *O*(1) |

Note: m and n are the width and height of the image, respectively, and k is the width of the filter kernel.

Compared to other algorithms, our visual information extraction operates at high speeds in an analog manner and eliminates the need for extra storage space.

## **Supplementary Table 3 Comparative Analysis of Our Methods with Current Neuromorphic Technologies**

1. Differential Neuromorphic Computing

Compared to existing neuromorphic technologies, our method stands out for its compatibility with multiple sensory modalities, the utilized neuromorphic device maturity, and its interoperability with conventional sensor and neuromorphic computing technologies.

2. Hardware Implementation with Memristor Arrays

In our demonstration, the memristive tactile system is realized through a hardware implementation, showcasing its efficiency; the visual information processing is conducted through simulations. Thus, a direct comparison of hardware implementation in visual information processing is not feasible. Instead, we focus on examining the data sources, decision-making features, and application scenarios utilized in our visual processing experiments. This comparison highlights the practicality of our method, illustrating its alignment with driving data and scenarios.

3. Bridging Sensory Processing with Decision-Making

In this aspect, we focus on low-level processing akin to mimicking biological tactile receptors and the subsequent generation of intermediate visual features crucial for decision-making. The effectiveness of these features in reducing time delays and enhancing decision accuracy is a key focus. For high-level decision-making, we provide insights into the movement control used in tactile experiments as illustrative examples.

|  | **This work** | **Lee et al.**(*63*) | **Lee et al.**(*64*) | **Zhang et al.**(*26*) | **Zhou et al.**(*65*) | **Liu et al.**(*11*) | **Jayachandran et al.**(*27*) | **John et al.**(*52*) | **Yuan et al.**(*66*) | **Huang et al.**(*67*) | **Li et al.**(*68*) |
| --- | --- | --- | --- | --- | --- | --- | --- | --- | --- | --- | --- |
| **Sensory modalities** | 4 types | 1 type | 2 types | 1 type | 1 type | 1 type | 1 type | 1 type | 4 types | 1 type | 1 type |
| **Neuromorphic**  **device maturity** | Commercial available | Lab  fabrication | Not  applicable | Lab  fabrication | Lab  fabrication | Lab  fabrication | Lab  fabrication | Lab  fabrication | Lab  fabrication | Lab  fabrication | Not  applicable |
| **Interoperability** | ^1^✔ | ✖ | ✔ | ✖ | ✖ | ✔ | ✖ | ✔ | ✔ | ✖ | ✖ |
|  | ^2^✔ | ✖ | ✔ | ✖ | ✖ | ✖ | ✖ | ✖ | ✖ | ✖ | ✖ |
| **Low-level processing functions** | Nociception | Proprioception | Slow adaptation | Not  applicable | Not  applicable | Nociception | Not  applicable | Nociception | Spike encoding | Not  applicable | Slow adaptation |
|  | Slow adaptation |  |  |  |  |  |  |  |  |  |  |
|  |  |  | Fast adaptation |  |  |  |  |  |  |  | Fast adaptation |
|  | Fast adaptation |  |  |  |  |  |  |  |  |  |  |
| **High-level decision-**  **making** | Pain  reflex | Voluntary motion | Slip detection | Not  applicable | Not applicable | Pain  reflex | Not applicable | Pain  reflex | Not applicable | Not  applicable | Slip detection |
|  | Slip detection |  |  |  |  |  |  |  |  |  |  |
| **Data sources** | Field-based data | Not  applicable | Not applicable | Laboratory  based data | Laboratory  based data | Not applicable | Laboratory  based data | Not applicable | Not applicable | Simulation | Not applicable |
| **Features**  **for decision-**  **making** | Relative motion | Not  applicable | Not applicable | Motion  target | Brightness  change | Not applicable | Approaching target | Not applicable | Not applicable | Historical trajectory | Not applicable |
|  | Brightness  change |  |  |  |  |  |  |  |  |  |  |
|  | Historical trajectory |  |  |  |  |  |  |  |  |  |  |
| **Scenarios** | Moving pedestrian | Not  applicable | Not applicable | Moving pedestrian | Waving hands | Not applicable | RC car approaching another car | Not applicable | Not applicable | Moving  light spots | Not applicable |
|  | Road signs |  |  |  |  |  |  |  |  |  |  |
|  | Warning light |  |  |  |  |  |  |  |  |  |  |
|  |  |  |  | Moving remote control (RC) car |  |  | RC car approaching wall |  |  |  |  |
|  | Overtaking |  |  |  |  |  |  |  |  |  |  |
|  | Taillight and five other scenarios |  |  |  |  |  |  |  |  |  |  |

Note: Interoperability^1^ addresses the system's capability to integrate and function with commercial standard sensors in place of the originally demonstrated one. Interoperability^2^ examines the system's flexibility to be adapted or replaced by different neuromorphic technologies.

**References**

1. Guan, T. *et al.* GA-Nav: Efficient Terrain Segmentation for Robot Navigation in Unstructured Outdoor Environments. *IEEE Robot. Autom. Lett.* **7**, 8138–8145 (2022).

2. Brosque, C., Galbally, E., Khatib, O., Fischer, M. "Human-Robot Collaboration in Construction: Opportunities and Challenges" in *2020 International Congress on Human-Computer Interaction, Optimization and Robotic Applications (HORA)* (2020), pp. 1–8. https://doi.org/10.1109/HORA49412.2020.9152888

3. Zhao, Y. *et al.* Twisting for soft intelligent autonomous robot in unstructured environments. *Proc Natl Acad Sci U S A* **119**, e2200265119 (2022).

4. M. Beetz, F. Bálint-Benczédi, N. Blodow, D. Nyga, T. Wiedemeyer, Z.-C. Márton, “RoboSherlock: Unstructured information processing for robot perception” in *2015 IEEE International Conference on Robotics and Automation (ICRA)* (2015), pp. 1549–1556.

5. T. G. Thuruthel, B. Shih, C. Laschi, M. T. Tolley, Soft robot perception using embedded soft sensors and recurrent neural networks. *Sci. Robot.* **4**, eaav1488 (2019).

6. A. Billard, D. Kragic, Trends and challenges in robot manipulation. *Science* **364**, eaat8414 (2019).

7. R. Deimel, O. Brock, A novel type of compliant and underactuated robotic hand for dexterous grasping. *The International Journal of Robotics Research* **35**, 161–185 (2016).

8. A. M. Dollar, R. D. Howe, Towards grasping in unstructured environments: grasper compliance and configuration optimization. *Adv. Robot.* **19**, 523–543 (2005).

9. B. Shih, D. Shah, J. Li, T. G. Thuruthel, Y.-L. Park, F. Iida, Z. Bao, R. Kramer-Bottiglio, M. T. Tolley, Electronic skins and machine learning for intelligent soft robots. *Sci. Robot.* **5**, eaaz9239 (2020).

10. F. Liu, S. Deswal, A. Christou, Y. Sandamirskaya, M. Kaboli, R. Dahiya, Neuro-inspired electronic skin for robots. *Sci. Robot.* **7**, eabl7344 (2022).

11. F. Liu, S. Deswal, A. Christou, M. Shojaei Baghini, R. Chirila, D. Shakthivel, M. Chakraborty, R. Dahiya, Printed synaptic transistor–based electronic skin for robots to feel and learn. *Sci. Robot.* **7**, eabl7286 (2022).

12. V. Ortenzi, M. Controzzi, F. Cini, J. Leitner, M. Bianchi, M. A. Roa, P. Corke, Robotic manipulation and the role of the task in the metric of success. *Nat Mach Intell* **1**, 340–346 (2019).

13. Z. Li, P. Zhao, C. Jiang, W. Huang, H. Liang, A Learning-Based Model Predictive Trajectory Planning Controller for Automated Driving in Unstructured Dynamic Environments. *IEEE Transactions on Vehicular Technology* **71**, 5944–5959 (2022).

14. H. Min, X. Xiong, P. Wang, Y. Yu, Autonomous driving path planning algorithm based on improved A* algorithm in unstructured environment. *Proceedings of the Institution of Mechanical Engineers, Part D: Journal of Automobile Engineering* **235**, 513–526 (2021).

15. Y. Qi, B. He, R. Wang, L. Wang, Y. Xu, Hierarchical Motion Planning for Autonomous Vehicles in Unstructured Dynamic Environments. *IEEE Robotics and Automation Letters* **8**, 496–503 (2023).

16. J.-Q. Yang, R. Wang, Y. Ren, J.-Y. Mao, Z.-P. Wang, Y. Zhou, S.-T. Han, Neuromorphic Engineering: From Biological to Spike-Based Hardware Nervous Systems. *Adv. Mater.* **32**, 2003610 (2020).

17. X. Xiao, J. Hu, S. Tang, K. Yan, B. Gao, H. Chen, D. Zou, Recent Advances in Halide Perovskite Memristors: Materials, Structures, Mechanisms, and Applications. *Adv. Mater. Technologies* **5**, 1900914 (2020).

18. K. Sun, J. Chen, X. Yan, The Future of Memristors: Materials Engineering and Neural Networks. *Adv. Funct. Mater.* **31**, 2006773 (2021).

19. Y. G. Song, J. M. Suh, J. Y. Park, J. E. Kim, S. Y. Chun, J. U. Kwon, H. Lee, H. W. Jang, S. Kim, C.-Y. Kang, J. H. Yoon, Artificial Adaptive and Maladaptive Sensory Receptors Based on a Surface-Dominated Diffusive Memristor. *Adv. Sci.* **9**, 2103484 (2022).

20. J. Ge, S. Zhang, Z. Liu, Z. Xie, S. Pan, Flexible artificial nociceptor using a biopolymer-based forming-free memristor. *Nanoscale* **11**, 6591–6601 (2019).

21. C. Zhang, W. B. Ye, K. Zhou, H.-Y. Chen, J.-Q. Yang, G. Ding, X. Chen, Y. Zhou, L. Zhou, F. Li, S.-T. Han, Bioinspired Artificial Sensory Nerve Based on Nafion Memristor. *Adv. Funct. Mater.* **29**, 1808783 (2019).

22. J. H. Yoon, Z. Wang, K. M. Kim, H. Wu, V. Ravichandran, Q. Xia, C. S. Hwang, J. J. Yang, An artificial nociceptor based on a diffusive memristor. *Nat Commun* **9**, 417 (2018).

23. Y. Kim, Y. J. Kwon, D. E. Kwon, K. J. Yoon, J. H. Yoon, S. Yoo, H. J. Kim, T. H. Park, J.-W. Han, K. M. Kim, C. S. Hwang, Nociceptive Memristor. *Adv. Mater.* **30**, 1704320 (2018).

24. S. Chen, Z. Lou, D. Chen, G. Shen, An Artificial Flexible Visual Memory System Based on an UV-Motivated Memristor. *Adv. Mater.* **30**, 1705400 (2018).

25. C. Jiang, Q. Li, N. Sun, J. Huang, R. Ji, S. Bi, Q. Guo, J. Song, A high-performance bionic pressure memory device based on piezo-OLED and piezo-memristor as luminescence-fish neuromorphic tactile system. *Nano Energy* **77**, 105120 (2020).

26. Z. Zhang, S. Wang, C. Liu, R. Xie, W. Hu, P. Zhou, All-in-one two-dimensional retinomorphic hardware device for motion detection and recognition. *Nat. Nanotechnol.* **17**, 27–32 (2022).

27. D. Jayachandran, A. Oberoi, A. Sebastian, T. H. Choudhury, B. Shankar, J. M. Redwing, S. Das, A low-power biomimetic collision detector based on an in-memory molybdenum disulfide photodetector. *Nat Electron* **3**, 646–655 (2020).

28. V. E. Abraira, D. D. Ginty, The Sensory Neurons of Touch. *Neuron* **79**, 618–639 (2013).

29. A. Lampert, D. L. Bennett, L. A. McDermott, A. Neureiter, E. Eberhardt, B. Winner, M. Zenke, Human sensory neurons derived from pluripotent stem cells for disease modelling and personalized medicine. *Neurobiology of Pain* **8**, 100055 (2020).

30. E. Kuehn, J. Dinse, E. Jakobsen, X. Long, A. Schäfer, P.-L. Bazin, A. Villringer, M. I. Sereno, D. S. Margulies, Body Topography Parcellates Human Sensory and Motor Cortex. *Cerebral Cortex* **27**, 3790–3805 (2017).

31. A. Handler, D. D. Ginty, The mechanosensory neurons of touch and their mechanisms of activation. *Nat Rev Neurosci* **22**, 521–537 (2021).

32. J. del Mármol, M. A. Yedlin, V. Ruta, The structural basis of odorant recognition in insect olfactory receptors. *Nature* **597**, 126–131 (2021).

33. J. A. Butterwick, J. Del Mármol, K. H. Kim, M. A. Kahlson, J. A. Rogow, T. Walz, V. Ruta, Cryo-EM structure of the insect olfactory receptor Orco. *Nature* **560**, 447–452 (2018).

34. N. L. Neubarth, A. J. Emanuel, Y. Liu, M. W. Springel, A. Handler, Q. Zhang, B. P. Lehnert, C. Guo, L. L. Orefice, A. Abdelaziz, M. M. DeLisle, M. Iskols, J. Rhyins, S. J. Kim, S. J. Cattel, W. Regehr, C. D. Harvey, J. Drugowitsch, D. D. Ginty, Meissner corpuscles and their spatially intermingled afferents underlie gentle touch perception. *Science* **368**, eabb2751 (2020).

35. L. L. Tan, R. Kuner, Neocortical circuits in pain and pain relief. *Nat Rev Neurosci* **22**, 458–471 (2021).

36. K. J. Blake, X. R. Jiang, I. M. Chiu, Neuronal Regulation of Immunity in the Skin and Lungs. *Trends in Neurosciences* **42**, 537–551 (2019).

37. J. Benda, Neural adaptation. *Current Biology* **31**, R110–R116 (2021).

38. C. R. Donnelly, O. Chen, R.-R. Ji, How Do Sensory Neurons Sense Danger Signals? *Trends in Neurosciences* **43**, 822–838 (2020).

39. F. Sun, Q. Lu, S. Feng, T. Zhang, Flexible Artificial Sensory Systems Based on Neuromorphic Devices. *ACS Nano* **15**, 3875–3899 (2021).

40. M. Wooten, H.-J. Weng, T. V. Hartke, J. Borzan, A. H. Klein, B. Turnquist, X. Dong, R. A. Meyer, M. Ringkamp, Three functionally distinct classes of C-fibre nociceptors in primates. *Nat Commun* **5**, 4122 (2014).

41. C.-W. Woo, L. Schmidt, A. Krishnan, M. Jepma, M. Roy, M. A. Lindquist, L. Y. Atlas, T. D. Wager, Quantifying cerebral contributions to pain beyond nociception. *Nat Commun* **8**, 14211 (2017).

42. A. E. Dubin, A. Patapoutian, Nociceptors: the sensors of the pain pathway. *J Clin Invest* **120**, 3760–3772 (2010).

43. M. S. Gold, G. F. Gebhart, Nociceptor sensitization in pain pathogenesis. *Nat Med* **16**, 1248–1257 (2010).

44. A. Zimmerman, L. Bai, D. D. Ginty, The gentle touch receptors of mammalian skin. *Science* **346**, 950–954 (2014).

45. A. Chortos, J. Liu, Z. Bao, Pursuing prosthetic electronic skin. *Nature Mater* **15**, 937–950 (2016).

46. L. Spillmann, J. S. Werner, *Visual Perception: The Neurophysiological Foundations* (Elsevier, 2012).

47. C.-H. Sung, J.-Z. Chuang, The cell biology of vision. *Journal of Cell Biology* **190**, 953–963 (2010).

48. D. Atchison, *Optics of the Human Eye* (CRC Press, Boca Raton, ed. 2, 2023).

49. A. I. Basbaum, D. M. Bautista, G. Scherrer, D. Julius, Cellular and Molecular Mechanisms of Pain. *Cell* **139**, 267–284 (2009).

50. R. S. Dahiya, G. Metta, M. Valle, G. Sandini, Tactile Sensing—From Humans to Humanoids. *IEEE Transactions on Robotics* **26**, 1–20 (2010).

51. L. E. Osborn, A. Dragomir, J. L. Betthauser, C. L. Hunt, H. H. Nguyen, R. R. Kaliki, N. V. Thakor, Prosthesis with neuromorphic multilayered e-dermis perceives touch and pain. *Sci. Robot.* **3**, eaat3818 (2018).

52. R. A. John, N. Tiwari, M. I. B. Patdillah, M. R. Kulkarni, N. Tiwari, J. Basu, S. K. Bose, Ankit, C. J. Yu, A. Nirmal, S. K. Vishwanath, C. Bartolozzi, A. Basu, N. Mathews, Self healable neuromorphic memtransistor elements for decentralized sensory signal processing in robotics. *Nat Commun* **11**, 4030 (2020).

53. M. Lee, G. J. Lee, H. J. Jang, E. Joh, H. Cho, M. S. Kim, H. M. Kim, K. M. Kang, J. H. Lee, M. Kim, H. Jang, J.-E. Yeo, F. Durand, N. Lu, D.-H. Kim, Y. M. Song, An amphibious artificial vision system with a panoramic visual field. *Nat Electron* **5**, 452–459 (2022).

54. Y. Chai, In-sensor computing for machine vision. *Nature* **579**, 32–33 (2020).

55. Y. Yang, C. Pan, Y. Li, X. Yangdong, P. Wang, Z.-A. Li, S. Wang, W. Yu, G. Liu, B. Cheng, Z. Di, S.-J. Liang, F. Miao, In-sensor dynamic computing for intelligent machine vision. *Nat Electron* **7**, 225–233 (2024).

56. D. Lee, M. Park, Y. Baek, B. Bae, J. Heo, K. Lee, In-sensor image memorization and encoding via optical neurons for bio-stimulus domain reduction toward visual cognitive processing. *Nat Commun* **13**, 5223 (2022).

57. Y. Wang, Y. Gong, S. Huang, X. Xing, Z. Lv, J. Wang, J.-Q. Yang, G. Zhang, Y. Zhou, S.-T. Han, Memristor-based biomimetic compound eye for real-time collision detection. *Nat Commun* **12**, 5979 (2021).

58. G. Zhou, J. Li, Q. Song, L. Wang, Z. Ren, B. Sun, X. Hu, W. Wang, G. Xu, X. Chen, L. Cheng, F. Zhou, S. Duan, Full hardware implementation of neuromorphic visual system based on multimodal optoelectronic resistive memory arrays for versatile image processing. *Nat Commun* **14**, 8489 (2023).

59. D. Weikersdorfer, D. B. Adrian, D. Cremers, J. Conradt, “Event-based 3D SLAM with a depth-augmented dynamic vision sensor” in *2014 IEEE International Conference on Robotics and Automation (ICRA)* (2014; https://ieeexplore.ieee.org/abstract/document/6906882), pp. 359–364.

60. C. Cabriel, T. Monfort, C. G. Specht, I. Izeddin, Event-based vision sensor for fast and dense single-molecule localization microscopy. *Nat. Photon.* **17**, 1105–1113 (2023).

61. G. Gallego, T. Delbrück, G. Orchard, C. Bartolozzi, B. Taba, A. Censi, S. Leutenegger, A. J. Davison, J. Conradt, K. Daniilidis, D. Scaramuzza, Event-Based Vision: A Survey. *IEEE Transactions on Pattern Analysis and Machine Intelligence* **44**, 154–180 (2022).

62. X. Zhang, Y. Zhuo, Q. Luo, Z. Wu, R. Midya, Z. Wang, W. Song, R. Wang, N. K. Upadhyay, Y. Fang, F. Kiani, M. Rao, Y. Yang, Q. Xia, Q. Liu, M. Liu, J. J. Yang, An artificial spiking afferent nerve based on Mott memristors for neurorobotics. *Nat Commun* **11**, 51 (2020).

63. Y. Lee, Y. Liu, D.-G. Seo, J. Y. Oh, Y. Kim, J. Li, J. Kang, J. Kim, J. Mun, A. M. Foudeh, Z. Bao, T.-W. Lee, A low-power stretchable neuromorphic nerve with proprioceptive feedback. *Nat. Biomed. Eng* **7**, 511–519 (2023).

64. W. W. Lee, Y. J. Tan, H. Yao, S. Li, H. H. See, M. Hon, K. A. Ng, B. Xiong, J. S. Ho, B. C. K. Tee, A neuro-inspired artificial peripheral nervous system for scalable electronic skins. *Sci. Robot.* **4**, eaax2198 (2019).

65. Y. Zhou, J. Fu, Z. Chen, F. Zhuge, Y. Wang, J. Yan, S. Ma, L. Xu, H. Yuan, M. Chan, X. Miao, Y. He, Y. Chai, Computational event-driven vision sensors for in-sensor spiking neural networks. *Nat Electron* **6**, 870–878 (2023).

66. R. Yuan, Q. Duan, P. J. Tiw, G. Li, Z. Xiao, Z. Jing, K. Yang, C. Liu, C. Ge, R. Huang, Y. Yang, A calibratable sensory neuron based on epitaxial VO2 for spike-based neuromorphic multisensory system. *Nat Commun* **13**, 3973 (2022).

67. P.-Y. Huang, B.-Y. Jiang, H.-J. Chen, J.-Y. Xu, K. Wang, C.-Y. Zhu, X.-Y. Hu, D. Li, L. Zhen, F.-C. Zhou, J.-K. Qin, C.-Y. Xu, Neuro-inspired optical sensor array for high-accuracy static image recognition and dynamic trace extraction. *Nat Commun* **14**, 6736 (2023).

68. S. Li, X. Chen, X. Li, H. Tian, C. Wang, B. Nie, J. He, J. Shao, Bioinspired robot skin with mechanically gated electron channels for sliding tactile perception. *Science Advances* **8**, eade0720 (2022).
