# Supplementary material for: Memristor-based adaptive neuromorphic perception in unstructured environments
Source: Nat Commun. 2024 May 31;15:4671. doi: 10.1038/s41467-024-48908-8 (PMC11143376; doi:10.1038/s41467-024-48908-8)
Supplement: Supplementary file 2 — Description of Supplementary Information [file 41467_2024_48908_MOESM2_ESM.pdf]

Description of Additional Supplementary Files

Supplementary Video SV1
Description: Grasping experiment 1: Sharp object

Supplementary Video SV2
Description: Grasping experiment 2: Slippery object

Supplementary Video SV3
Description: Visual information processing at night

Supplementary Video SV4
Description: Visual information processing in daytime

Supplementary Video SV5
Description: Visual information processing in various driving scenarios

Supplementary Video SV6
Description: Introduction to differential neuromorphic computing
